# A Walk into the LuxR Regulators of Actinobacteria: Phylogenomic Distribution and Functional Diversity

**DOI:** 10.1371/journal.pone.0046758

**Published:** 2012-10-08

**Authors:** Catarina Lopes Santos, Margarida Correia-Neves, Pedro Moradas-Ferreira, Marta Vaz Mendes

**Affiliations:** 1 IBMC - Instituto de Biologia Molecular e Celular, Universidade do Porto, Porto, Portugal; 2 Life and Health Sciences Research Institute (ICVS), School of Health Sciences, University of Minho, Campus de Gualtar, Braga, Portugal; 3 ICVS/3B’s - PT Government Associate Laboratory, Braga/Guimarães, Portugal; 4 ICBAS – Instituto de Ciências Biomédicas Abel Salazar, Universidade do Porto, Porto, Portugal; J. Craig Venter Institute, United States of America

## Abstract

LuxR regulators are a widely studied group of bacterial helix-turn-helix (HTH) transcription factors involved in the regulation of many genes coding for important traits at an ecological and medical level. This regulatory family is particularly known by their involvement in quorum-sensing (QS) mechanisms, i.e., in the bacterial ability to communicate through the synthesis and binding of molecular signals. However, these studies have been mainly focused on Gram-negative organisms, and the presence of LuxR regulators in the Gram-positive Actinobacteria phylum is still poorly explored. In this manuscript, the presence of LuxR regulators among Actinobacteria was assayed using a domain-based strategy. A total of 991 proteins having one LuxR domain were identified in 53 genome-sequenced actinobacterial species, of which 59% had an additional domain. In most cases (53%) this domain was REC (receiver domain), suggesting that LuxR regulators in Actinobacteria may either function as single transcription factors or as part of two-component systems. The frequency, distribution and evolutionary stability of each of these sub-families of regulators was analyzed and contextualized regarding the ecological niche occupied by each organism. The results show that the presence of extra-domains in the LuxR-regulators was likely driven by a general need to physically uncouple the signal sensing from the signal transduction. Moreover, the total frequency of LuxR regulators was shown to be dependent on genetic, metabolic and ecological variables. Finally, the functional annotation of the LuxR regulators revealed that the bacterial ecological niche has biased the specialization of these proteins. In the case of pathogens, our results suggest that LuxR regulators can be involved in virulence and are therefore promising targets for future studies in the health-related biotechnology field.

## Introduction

The LuxR family of DNA-binding proteins is characterized by the presence of a specific regulatory helix-turn-helix (HTH) domain, named LuxR, in the C-terminal region. The first protein of this family to be described was involved in the quorum-sensing (QS) circuit of the symbiotic organism *Vibrio fischeri*, being the transcriptional activator of its luminescence operon [1]. Gram-negative LuxR-type regulators involved in QS are known to be transcription factors that become activated upon sensing specific signals, usually acyl-homoserine lactones (AHLs), modulating the expression of their target genes [2], [3]. These QS-related LuxR-type proteins are composed of two different modules: the N-terminal region senses and/or binds their specific QS signal, whereas the C-terminal contains a conserved HTH motif that binds DNA and promotes gene expression/repression. LuxI is the synthase responsible for synthesizing the AHLs, and is therefore another key element in these QS circuits [2], [3]. Although *luxI* and its cognate *luxR* are frequently located in adjacent genome positions, suggesting instances of co-evolution, their distribution among bacteria is mostly discontinuous and marked by duplications, gene loss and HGT (horizontal gene transfer) events [4]. In fact, it is usual to find organisms with several pairs of *luxI*/*luxR*, each one inherited from a different source, demonstrating that this particular QS system is quite flexible in evolutionary terms [5], [6].

The classical LuxR-based QS mechanism described above for Gram-negative organisms is somewhat different from that observed in the Gram-positives. In fact, and instead of the Gram-negative single transcription factors, the two-component systems (TCS) appear to play a crucial role in the QS signaling of Gram-positives ([7] and cited references). Whereas in most cases the QS signals of Gram-negative organisms (AHLs) are internalized by passive diffusion [8], that does not seem to be the case in Gram-positive bacteria. Either due to the different nature of the QS signals, or due to the specificities of the Gram-positive cell wall, QS signals in these organisms usually require a dedicated exporter system (such as an ABC transporter) and cannot passively enter the surrounding cells [7]. TCS allow the bacteria to overcome this limitation, by physically uncoupling the signal sensor (a membrane-associated histidine protein kinase - HPK) from the response regulator – RR [9]. Adding to this, the nature of the QS signals is also different in the Gram-positive when compared to the Gram-negative organisms. In fact, no AHLs are known to act on the Gram-positive QS systems, where signaling is generally assured by cyclic or modified peptides and γ-butyrolactones (GBLs). Interestingly, and opposite to these lineage-specific signals, the so-called auto-inducer 2 (AI-2, furanosyl borate diester) is produced and sensed by a wide-spread group of bacteria, including Gram-positive and Gram-negative microorganisms [10]–[12].

The characterization of the first LuxR protein led to the elucidation of QS mechanisms and represented a turning point in the paradigmatic vision of bacterial colonies as cell aggregates. But besides QS in its *sensu stricto*, i.e., signal-mediated communication between single-species bacterial populations, LuxR-type proteins are known to be involved in a number of other cellular signaling pathways. In fact, there are LuxR regulators that are able to sense and respond to molecules produced by other bacterial species or eukaryotic organisms [4].In the Gram-negative bacteria, these regulators are considered to be “solo” or “orphan” *luxR* genes, i.e., *luxR* genes not associated with a *luxI*, and are currently viewed as a bacterial strategy to expand their regulatory network [4], [13]. Specifically the inter-kingdom communication has been shown in many instances to be crucial for the development of bacterial-eukaryotic relationships, as the LuxR regulators are responsible for modulating virulence factors expression, biofilms formation and even the hosts’ immune response [8]. Moreover, LuxR regulators may also be involved in intracellular signaling, as it happens for instance in the antibiotic biosynthesis by *Streptomyces* species [14].

The important role of LuxR domains in signaling mechanisms has prompted us to address the phylogenomic distribution and functional diversity of LuxR proteins in the heterogeneous Actinobacteria phylum, one of the largest groups of organisms in the bacterial kingdom. It should be highlighted that, although LuxR regulators, namely those associated with QS, have been widely addressed in many Gram-negative models, its presence in the Gram-positive Actinobacteria phylum has not been described at the same extent, and consequently the importance of LuxR regulators in these and other processes remains largely unknown. With the exception of the well characterized butyrolactone-based system of *Streptomyces* spp. [15], communication in this phylum has been scarcely explored and relies mostly on indirect evidence. In this context, this manuscript entails an extensive search and *in silico* characterization of all LuxR regulators in Actinobacteria. Our research revealed a diversified and stereoscopic organization of LuxR proteins among members of this phylum. In fact, not only have the original LuxR-encoding genes suffered a series of duplications presumably followed by functional specification, but they have also acquired different domains, originating new sub-families implicated in a wide range of functionalities.

## Results and Discussion

### Census on LuxR Domains of Actinobacteria

**Figure 1:**
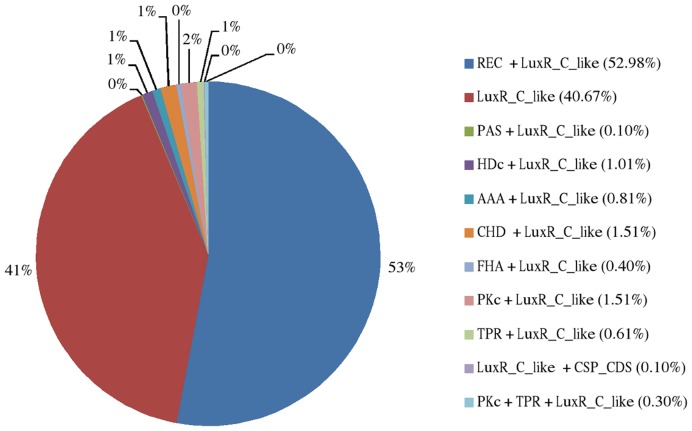
Distribution of the LuxR-containing sequences retrieved from Actinobacteria according to their domain architecture. REC, receiver domain; PAS, Per (period circadian protein), Arnt (Ah receptor nuclear translocator protein), Sim (single-minded protein); HDc, phosphohydrolase; AAA, ATPases associated with diverse cellular activities; CHD, cyclase homology domain; FHA, forkhead-associated domain; PKc, protein kinases catalytic domain; TPR, tetratricopeptide repeat domain; CSP_CDS, cold-shock protein with a S1-like cold shock domain.

In order to have an overview of the number and distribution of LuxR regulators (i.e., proteins containing a LuxR domain) in Actinobacteria, a domain-based dual approach was applied to the complete proteomes of a set of actinobacterial organisms chosen in order to represent the diversity of Actinobacteria in terms of phylogenetic groups, morphological types, ecological niches and metabolic abilities. A total of 991 protein sequences containing at least one LuxR domain were identified among 53 species (Table 1). These sequences are not evenly distributed among species, ranging from organisms with a single sequence (e.g., *Mycobacterium leprae*) to others with over 50 (e.g. *Streptomyces* spp.). Moreover, 59% of these proteins have an extra domain in addition to the LuxR (Table 1 and Fig. 1).

**Table 1:** Species considered in this study and their frequency of each sub-family of LuxR regulators.

Organism (total n° of seq)	Architecture	N° of seq
*Acidothermus cellulolyticus* 11B (6)	REC (cd00156) + LuxR_C_like (cd06170)	6
*Arthrobacter aurescens* TC1 (16)	REC (cd00156) + LuxR_C_like (cd06170)	5
	LuxR_C_like (cd06170)	11
*Arthrobacter chlorophenolicus* A6 (17)	REC (cd00156) + LuxR_C_like (cd06170)	5
	LuxR_C_like (cd06170)	11
	AAA (cd00009) + LuxR_C_like (cd06170)	1
*Arthrobacter* sp. FB24 (14)	REC (cd00156) + LuxR_C_like (cd06170)	5
	LuxR_C_like (cd06170)	9
*Bifidobacterium adolescentis* ATCC15703 (7)	REC (cd00156) + LuxR_C_like (cd06170)	7
*Bifidobacterium animalis* subsp. *lactis* AD011 (5)	REC (cd00156) + LuxR_C_like (cd06170)	5
*Bifidobacterium longum* DJO10A (4)	REC (cd00156) + LuxR_C_like (cd06170)	4
*Bifidobacterium longum* subsp. *infantis* ATCC15697 (14)	REC (cd00156) + LuxR_C_like (cd06170)	14
*Clavibacter michiganensis* subsp. *michiganensis* NCPPB 382 (17)	REC (cd00156) + LuxR_C_like (cd06170)	13
	LuxR_C_like (cd06170)	4
*Clavibacter michiganensis* subsp. *sepedonicus* (15)	REC (cd00156) + LuxR_C_like (cd06170)	10
	LuxR_C_like (cd06170)	5
*Corynebacterium aurimucosum* ATCC 700975 (8)	REC (cd00156) + LuxR_C_like (cd06170)	7
	LuxR_C_like (cd06170)	1
*Corynebacterium diphtheriae* NCTC 13129 (6)	REC (cd00156) + LuxR_C_like (cd06170)	5
	LuxR_C_like (cd06170)	1
*Corynebacterium efficiens* YS-314 (10)	REC (cd00156) + LuxR_C_like (cd06170)	6
	LuxR_C_like (cd06170)	4
*Corynebacterium glutamicum* R (8)	REC (cd00156) + LuxR_C_like (cd06170)	5
	LuxR_C_like (cd06170)	3
*Corynebacterium jeikeium* K411 (4)	REC (cd00156) + LuxR_C_like (cd06170)	3
	LuxR_C_like (cd06170)	1
*Corynebacterium urealyticum* DSM 7109 (4)	REC (cd00156) + LuxR_C_like (cd06170)	3
	LuxR_C_like (cd06170)	1
*Frankia alni* ACN14a (25)	REC (cd00156) + LuxR_C_like (cd06170)	13
	LuxR_C_like (cd06170)	12
*Frankia* sp. CcI3 (18)	REC (cd00156) + LuxR_C_like (cd06170)	6
	LuxR_C_like (cd06170)	12
*Frankia* sp. EAN1pec (48)	REC (cd00156) + LuxR_C_like (cd06170)	31
	LuxR_C_like (cd06170)	16
	AAA (cd00009) + LuxR_C_like (cd06170)	1
*Kineococcus radiotolerans* SRS30216 (28)	REC (cd00156) + LuxR_C_like (cd06170)	18
	LuxR_C_like (cd06170)	10
*Kocuria rhizophila* DC2201 (8)	REC (cd00156) + LuxR_C_like (cd06170)	7
	LuxR_C_like (cd06170)	1
*Leifsonia xyli* subsp. *xyli* str. CTCB07 (14)	REC (cd00156) + LuxR_C_like (cd06170)	7
	LuxR_C_like (cd06170)	7
*Mycobacterium abscessus* ATCC 19977 (9)	REC (cd00156) + LuxR_C_like (cd06170)	6
	LuxR_C_like (cd06170)	3
*Mycobacterium avium* 104 (8)	REC (cd00156) + LuxR_C_like (cd06170)	4
	LuxR_C_like (cd06170)	2
	HDc (cd00077) + LuxR_C_like (cd06170)	1
	AAA (cd00009) + LuxR_C_like (cd06170)	1
*Mycobacterium bovis* BCG str. Pasteur 1173P2 (7)	REC (cd00156) + LuxR_C_like (cd06170)	2
	LuxR_C_like (cd06170)	2
	CHD (cd07302) + LuxR_C_like (cd06170)	3
*Mycobacterium bovis* BCG str. Tokyo 172 (7)	REC (cd00156) + LuxR_C_like (cd06170)	2
	LuxR_C_like (cd06170)	2
	CHD (cd07302) + LuxR_C_like (cd06170)	3
*Mycobacterium gilvum* PYR-GCK (15)	REC (cd00156) + LuxR_C_like (cd06170)	6
	LuxR_C_like (cd06170)	8
	HDc (cd00077) + LuxR_C_like (cd06170)	1
*Mycobacterium leprae* Br4923 (1)	LuxR_C_like (cd06170)	1
*Mycobacterium leprae* TN (1)	LuxR_C_like (cd06170)	1
*Mycobacterium marinum* M (11)	REC (cd00156) + LuxR_C_like (cd06170)	5
	LuxR_C_like (cd06170)	4
	CHD (cd07302) + LuxR_C_like (cd06170)	2
*Mycobacterium smegmatis* str. MC2 155 (32)	REC (cd00156) + LuxR_C_like (cd06170)	13
	LuxR_C_like (cd06170)	18
	AAA (cd00009) + LuxR_C_like (cd06170)	1
*Mycobacterium* sp. JLS (21)	REC (cd00156) + LuxR_C_like (cd06170)	7
	LuxR_C_like (cd06170)	12
	HDc (cd00077) + LuxR_C_like (cd06170)	1
	AAA (cd00009) + LuxR_C_like (cd06170)	1
*Mycobacterium* sp. KMS (18)	REC (cd00156) + LuxR_C_like (cd06170)	7
	LuxR_C_like (cd06170)	10
	HDc (cd00077) + LuxR_C_like (cd06170)	1
*Mycobacterium* sp. MCS (18)	REC (cd00156) + LuxR_C_like (cd06170)	7
	LuxR_C_like (cd06170)	10
	HDc (cd00077) + LuxR_C_like (cd06170)	1
*Mycobacterium tuberculosis* H37Ra (7)	REC (cd00156) + LuxR_C_like (cd06170)	2
	LuxR_C_like (cd06170)	2
	CHD (cd07302) + LuxR_C_like (cd06170)	3
*Mycobacterium tuberculosis* H37Rv (7)	REC (cd00156) + LuxR_C_like (cd06170)	2
	LuxR_C_like (cd06170)	2
	CHD (cd07302) + LuxR_C_like (cd06170)	3
*Mycobacterium ulcerans* Agy99 (5)	REC (cd00156) + LuxR_C_like (cd06170)	3
	LuxR_C_like (cd06170)	2
*Mycobacterium vanbaalenii* PYR-1 (30)	REC (cd00156) + LuxR_C_like (cd06170)	11
	LuxR_C_like (cd06170)	16
	HDc (cd00077) + LuxR_C_like (cd06170)	1
	AAA(cd00009) + LuxR_C_like (cd06170)	1
	CHD (cd07302) + LuxR_C_like (cd06170)	1
*Nocardia farcinica* (28)	REC (cd00156) + LuxR_C_like (cd06170)	14
	LuxR_C_like (cd06170)	13
	FHA (cd00060) + LuxR_C_like (cd06170)	1
*Nocardioides* sp. JS614 (27)	REC (cd00156) + LuxR_C_like (cd06170)	14
	LuxR_C_like (cd06170)	11
	HDc (cd00077) + LuxR_C_like (cd06170)	2
*Propionibacterium acnes* KPA171202 (6)	REC (cd00156) + LuxR_C_like (cd06170)	6
*Renibacterium salmoninarum* ATCC 33209 (9)	REC (cd00156) + LuxR_C_like (cd06170)	4
	LuxR_C_like (cd06170)	5
*Rhodococcus erythropolis* PR4 (33)	REC (cd00156) + LuxR_C_like (cd06170)	20
	LuxR_C_like (cd06170)	12
	PKc (cd00180) + TPR (cd00189) + LuxR_C_like (cd06170)	1
*Rhodococcus opacus* B4 (50)	REC (cd00156) + LuxR_C_like (cd06170)	17
	LuxR_C_like (cd06170)	23
	PKc (cd00180) + LuxR_C_like (cd06170)	6
	HDc (cd00077) + LuxR_C_like (cd06170)	1
	FHA (cd00060) + LuxR_C_like (cd06170)	1
	TPR (cd00189) + LuxR_C_like (cd06170)	1
	LuxR_C_like (cd06170) + CSP_CDS(cd04458)	1
*Rhodococcus* sp. RHA1 (57)	REC (cd00156) + LuxR_C_like (cd06170)	17
	LuxR_C_like (cd06170)	24
	PKc (cd00180) + LuxR_C_like (cd06170)	9
	HDc (cd00077) + LuxR_C_like (cd06170)	1
	FHA (cd00060) + LuxR_C_like (cd06170)	2
	TPR (cd00189) + LuxR_C_like (cd06170)	2
	PKc (cd00180) + TPR (cd00189) + LuxR_C_like (cd06170)	2
*Rubrobacter xylanophilus* DSM 9941 (13)	REC (cd00156) + LuxR_C_like (cd06170)	8
	LuxR_C_like (cd06170)	4
	PAS (cd00130) + LuxR_C_like (cd06170)	1
*Saccharopolyspora erythraea* NRRL 2338 (52)	REC (cd00156) + LuxR_C_like (cd06170)	23
	LuxR_C_like (cd06170)	28
	TPR (cd00189) + LuxR_C_like (cd06170)	1
*Salinispora arenicola* CNS-205 (20)	REC (cd00156) + LuxR_C_like (cd06170)	7
	LuxR_C_like (cd06170)	13
*Salinispora tropica* CNB-440 (18)	REC (cd00156) + LuxR_C_like (cd06170)	9
	LuxR_C_like (cd06170)	9
*Streptomyces avermitilis* MA-4680 (50)	REC (cd00156) + LuxR_C_like (cd06170)	32
	LuxR_C_like (cd06170)	18
*Streptomyces coelicolor* A3(2) (71)	REC (cd00156) + LuxR_C_like (cd06170)	45
	LuxR_C_like (cd06170)	24
	AAA (cd00009) + LuxR_C_like (cd06170)	2
*Streptomyces griseus* subsp. *griseus* NBRC 13350 (48)	REC (cd00156) + LuxR_C_like (cd06170)	36
	LuxR_C_like (cd06170)	11
	TPR (cd00189) + LuxR_C_like (cd06170)	1
*Thermobifida fusca* YX (16)	REC (cd00156) + LuxR_C_like (cd06170)	11
	LuxR_C_like (cd06170)	4
	TPR (cd00189) + LuxR_C_like (cd06170)	1

Most of the subfamilies in which the LuxR domain appears associated with an N-terminal domain have a low frequency, with the exception of the REC+LuxR group (Fig. 1). Although varied, all these extra-domains share a common feature: they all have a more or less direct relationship with signal transduction, among other functions. This suggests that these combinations of domains result in proteins related with the modulation of genetic expression through signal perception.

Among the LuxR-associated domains, REC domain (receiver, a CheY-like phosphoacceptor) should be highlighted due to its particularly high frequency: in fact, REC appears associated to LuxR in 53% of all retrieved LuxR protein sequences (Fig. 1). REC is an evolutionary stable structural unit that is part of more than 70,000 proteins classified into 1,716 different architectures [16]. Being mainly related with signal transduction, REC’s presence upstream LuxR domains suggest that LuxR proteins should not be viewed only as single transcriptions factors, as is usually the case in Gram-negative QS models, but also as part of TCS, typical in Gram-positive signaling. This seems to be the case of the REC+LuxR proteins, which most likely constitute RRs of actinobacterial TCS. This specific association REC+LuxR, which appears to be very common in Actinobacteria, has already been described, for instance, in the QS-related competence regulation of *Bacillus subtilis* and *Lactobacillus plantarum* [9].

Overall, the LuxR family of proteins in Actinobacteria include two major subfamilies: one that resembles the classical LuxR transcriptional regulators from Gram-negative organisms, having a single specific hit in the CD-search - the LuxR domain - and probably constituting one-component transcriptional regulators; and another in which the LuxR domain is associated with a N-terminal REC domain. In a third and smaller group of sequences, LuxR domain appears associated with a series of signal transduction-related domains other than REC, forming multidomain proteins that may also be part of QS-related TCS circuits, although additional or complementary functions should not be disregarded.

A similar search to the one performed for LuxR regulators yielded almost no hits for LuxI proteins in Actinobacteria. Classical LuxI proteins have in common the Autoind_synth domain (PF00765). However, searches conducted in the Pfam database regarding this domain in Actinobacteria yielded a single hit, belonging to *Streptomyces sviceus* (SSEG_02829). Further BlastP searches were conducted in the NCBI, but no other occurrences of LuxI homologues in Actinobacteria were reported. This indicates that the retrieved LuxR-containing sequences are not associated with a specific LuxI synthase, which is in agreement with the fact that no AHLs or AHL-sensing systems have been described for Gram-positive organisms so far. Therefore, if involved in QS, these proteins are likely specialized in sensing stimulus different from AHLs. Alternatively these regulators may be involved in transducing signals produced by other bacterial species or eukaryotic hosts [4], [13], or even in intracellular signaling pathways.

### The Phylogenetic History of the LuxR Proteins

**Figure 2:**
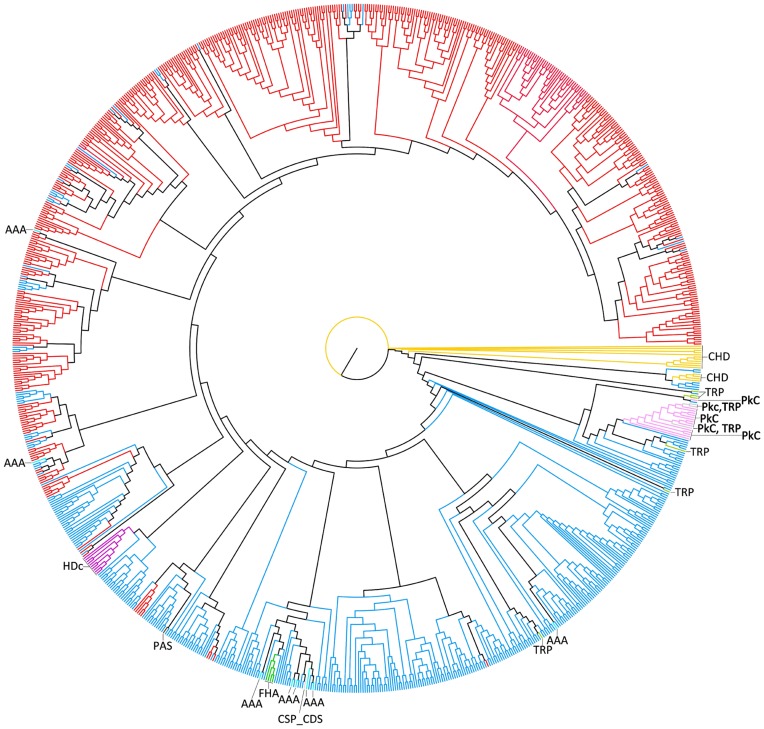
Neighbor-Joining unrooted tree of all LuxR-containing sequences retrieved from Actinobacteria. The branches colour refers to each protein architecture: LuxR are coloured in blue; REC+LuxR are coloured in red; CHD+LuxR are coloured in yellow; TPR+LuxR are coloured in light green; Pkc+LuxR are coloured in light pink; AAA+LuxR are coloured in light blue; LuxR+CSP_CDS are coloured in light red; FHA+LuxR are coloured in green; HDc+LuxR are coloured in pink; PAS+LuxR are coloured in brown; Pkc+TPR+LuxR are coloured in grey. With the exception of REC, the presence of these extra domains is highlighted in the figure.

**Figure 3:**
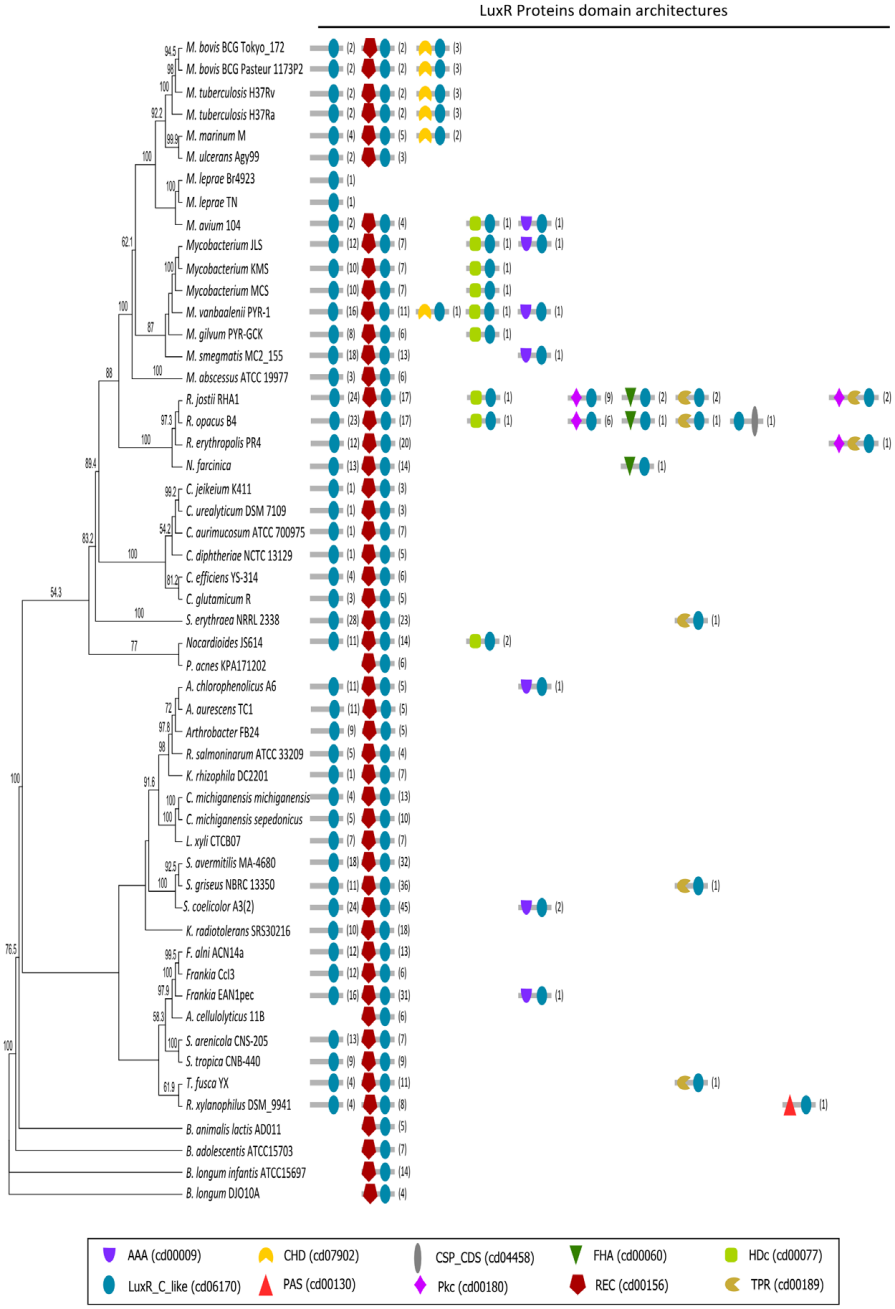
Neighbor-Joining unrooted tree of all actinobacterial species considered in this study. The domain composition and number of the LuxR proteins’ family in each species are mapped on the right.

The diversity of domain compositions and the broad distribution of the LuxR proteins in Actinobacteria raised the question of their phylogenetic history. To elucidate this point, a phylogenetic tree of all 991 LuxR proteins was built and analyzed in terms of distribution of domain architectures (Fig. 2). To detect duplications, deletions and HGT events, this tree was compared to a Neighbor-Joining (NJ) Actinobacteria species tree, based upon their 16S rRNA gene sequences (Fig. 3). To validate this Actinobacteria phylogeny, a Maximum Likelihood (ML) tree for the 16S sequences was also computed (Fig. S1). This tree is mostly coherent with that built with the NJ algorithm, supporting our results. Moreover, both trees are generally consistent with the literature, although the positioning of *Bifidobacterium* and *Propionibacterium acnes* is still not well resolved. In fact, and regarding *P. acnes*, one can find in the literature instances where it appears as a deep-branching lineage, or, alternatively, in a cluster with *Nocardioides* spp., as is the case in the present study [17], [18]. Moreover, *Bifidobacterium* genus sometimes appears in the basis of the actinobacterial tree, as in Fig. 3 and S1, but it may also appear as a sister cluster to Actinomycetales and Micrococcales [17], [18]. As so, and to avoid erroneous conclusions, phylogenetic inferences on the LuxR proteins were restricted to actinobacterial clusters with high bootstrap support and consistent between both NJ and ML trees and with previously published phylogenetic studies.

The analysis of both trees (Fig. 2 and Fig. 3) suggests that the ancestor gene sequence codified for a protein with a single LuxR domain. This ancestor gene was maintained in the majority of the organisms, being lost only in *P. acnes, Acidothermus cellulolyticus* and *Bifidobacterium* spp. (Fig. 3). Regarding the appearance of the sub-family of proteins including a REC domain in the N-terminal region, the most parsimonious explanation involves a single gene fusion occurring in the ancestor of all organisms considered. After speciation, this event translated into the phylogenetic division of the LuxR family into two main architecture-defined groups - the LuxR and REC+LuxR - forming the two main branches in the LuxR-tree (Fig. 2). Notwithstanding, the adaptation of each species to its ecological niche led to a series of lineage-specific gene fusions, fissions and acquisitions that appear as isolated clusters of a given domain architecture interspersed among other architecture. In fact, it is possible to visualize 18 occurrences of REC+LuxR proteins among the single-domain group (distributed in 3 monophyletic groups and 5 isolated sequences) and 49 single-domain proteins among the REC+LuxR group (distributed in 10 monophyletic groups and 17 isolated sequences). The origin of these sequences can be explained by HGT events, particularly when the sequences appear isolated, and independent gene fusions/fissions, when the sequences are clustered in monophyletic groups. Although the data available does not offer a solid ground to choose one of the hypothesis over the other, it should be highlighted that gene expansion and HGT are considerably common among TCS members [19]. Moreover, HGT is known to have an important role in the transfer of domain fusions [20].

Regarding the smaller sub-families of LuxR proteins, almost all of them emerge from single-domain LuxR protein branches, suggesting that they arose by specific gene fusions between the ancestor gene and other domain-codifying genes. The limited distribution of these smaller sub-families suggests that their fixation in the populations was driven by specific selective pressures whose influence was exclusively present or stronger in certain taxonomical groups. The Pkc+LuxR sub-family is exclusive of *Rhodococcus* spp, as well as the only sub-family of 3-domain proteins, the Pkc+TPR+LuxR (Fig. 3). Interestingly, 14 out of 15 members of the Pkc+LuxR and the 3 members of Pkc+TPR+LuxR are located in a monophyletic group with a considerable strong bootstrap support (73.8%), whereas the last Pkc+LuxR element appears in a closely related group (Fig. 2). This result suggests that the architecture Pkc+TRP+LuxR was formed by a fusion of the TRP domain to the already existent Pkc+LuxR. This subfamily, in its turn, appears to have been formed in the *Rhodococcus* spp. ancestor, suffering a species-specific gene expansion after this group speciation, which led to the different number of Pkc+LuxR sequences in the different *Rhodococcus* species. The presence of one homolog outside the monophyletic group can be explained by the need of the duplicated genes to specialize into specific functionalities to overcome redundancy and avoid deletion [21]. The FHA+LuxR subfamily members are clustered in a monophyletic group with 62.9% of bootstrap support (Fig. 2). The distribution of these proteins in the studied organisms suggests that the gene coding for this sub-family arose through a single FHA and LuxR gene fusion in the common ancestor of *Rhodococcus* spp. and *Nocardia* spp., and was later lost from *Rhodococcus erythropolis* (Fig. 3). All the proteins with an HDc+LuxR domain architecture also clustered together in a monophyletic group with 99.2% of bootstrap support (Fig. 2). However, their distribution pattern among Actinobacteria species (Fig. 3) raises two different hypotheses regarding their origin: either the HDc and LuxR gene fission occurred only once in the common ancestor of all species that possess it; or the HDc acquisition occurred in the common ancestor of *Rhodococcus* spp., *Mycobacterium* spp. and *Nocardia* spp., followed by an HGT event to *Nocardioides*. Both hypotheses imply that a certain number of species have lost the HDc+LuxR codifying gene: 20 in the first case and 12 in the second, which, from a more parsimonious point of view, can be explained by 9 and 6 independent loss events, respectively. Finally, there are 15 proteins with the CHD+LuxR architecture, all of them clustered in a monophyletic group that also includes 6 LuxR single-domain proteins from *Mycobacterium* spp., with a bootstrap support of 98.1% (Fig. 2). According to their distribution in the species tree (Fig. 3), the origin of this group was either in the common ancestor of the *Mycobacterium tuberculosis* complex group, and later spread to *Mycobacterium marinum* and *Mycobacterium vanbaalenii* by HGT; or it arose on the ancestor common to *M. tuberculosis* complex, *M. marinum* and *Mycobacterium ulcerans*, was later lost from *M. ulcerans* and spread to *M. vanbaalenii* by HGT.

The analysis of the AAA+LuxR and the TRP+LuxR codifying genes resulted in more complex phylogenetic histories. The AAA+LuxR proteins of *Mycobacterium* spp. form a monophyletic group with LuxR single-domain proteins and a LuxR+CSP_CSD protein (bootstrap: 56.1%), and therefore are likely to have a common origin (Fig. 2). However, 4 other AAA+LuxR elements belonging to other species are spread among the LuxR tree and are heterogeneously distributed among the species tree (Fig. 3). This suggests that either the gene codifying for this protein had multiple origins, or it had a single origin and got dispersed through HGT events. This last hypothesis is supported by the fact that all the species that have this protein share the soil as an habitat. Interestingly, the only instances in which a multidomain protein is more closely associated to REC+LuxR proteins than to single domain LuxR proteins are two AAA+LuxR members (Frean1_3551 and SCO4263). The TRP+LuxR case is similar: of the 6 elements in this family, only 2 (from *Rhodococcus* spp.) cluster in a monophyletic group, with 100% bootstrap support, whereas the others are spread among the LuxR tree (Fig. 2). Again, all the species that have proteins from this sub-family may be found in the soil (Fig. 3), therefore facilitating potential HGT events. Finally, both LuxR+CSP_CSD and PAS+LuxR occur only once, and both are closely related to a single LuxR domain protein.

To confirm and validate the results obtained from this phylogenetic analysis, an ML tree of the LuxR family members was constructed and analyzed (Fig. S2). Overall, the occurrence of the two main sub-families (single domain LuxR and REC+LuxR proteins), marked by the punctual incidence of HGT and independent gene fusion/fission events, is maintained, although the number of events needed to explain the phylogenetic separation of these subfamilies varies. Furthermore, the individual phylogenetic histories and the statistics supporting the hypothetical phylogenetic histories of the smaller LuxR sub-families are sustained. The only exceptions are the positions of the Frean1_3551 and the SCO4263 (AAA+LuxR proteins) which, in this tree, appear closely associated with LuxR single-domain proteins, instead of REC+LuxR proteins as it happens in the NJ tree.

The phylogenetic results described suggest a conspicuous promiscuity of the LuxR domain among Actinobacteria. In fact, this domain appears associated to a number of different signal-transduction related domains, conveying its role as the effector of RR in TCSs. The fact that neither LuxR nor the associated domains are exclusive implies that these RRs, as many others, are likely to have evolved from a fusion of two previously independent domains, a route of evolution which is now widely accepted as a major source of genome innovation [20]. One of the adaptive advantages of domain fusion, considering that fusion happens when those domains are somehow linked functionally, is the tight correlation of their gene expression [20]. Moreover, and particularly important in the case of the TCS, the domain fusion allows an increase of efficiency since the signal transduction step becomes linked with the corresponding biochemical reaction [20]. Interestingly, a previously published study focused on the phylogeny of the histidine kinase domain of the HPKs, the RRs partners in TCS, has unveiled that lineage-specific expansions and HGT have played a fundamental role. Moreover, it has shown that whereas after HGT the HPKs were likely to maintain the same domain architecture, after lineage-specific expansions the histidine kinases were likely to associate with signaling domains other than the original ones [19]. Being the HPK strongly associated to their specific RRs, one could speculate that the same processes occurred in the RRs. Notwithstanding, this study reveals that cases such *Streptomyces*, in which an extensive lineage-specific expansion of the LuxR family clearly occurred, have a very limited diversity of associated domains. These results suggest that the novel genetic diversity originated by the LuxR specific lineage-expansions is mostly at the level of point mutations instead of domains re-shuffling. Nevertheless, it should be taken into account the fact that the study by Alm and collaborators [19] focused on the entire group of HPKs, whereas this study focused on a particular group of RRs – those that have LuxR as an effector domain. Therefore, given that it is a more specific, and consequently more recent, family of proteins, the LuxR group may be in the initial steps of diversification and specialization, and domain re-shuffling may follow this initial phase of punctual sequence mutations.

### The Distribution of the LuxR Family According to Genetic, Ecological and Metabolic Variables

To understand which evolutionary pressures have shaped the heterogeneous distribution of the LuxR family in Actinobacteria, all the 53 organisms under analysis were classified according to a series of genetic, ecological and metabolic variables (Table S1). Two different correlation tests, the Spearman Rank Order Correlation and the Eta coefficient, were employed to detect the presence of simultaneous variation between the organisms classification, the frequency of the different LuxR proteins architecture, and the total number of sequences having a LuxR domain. The statistically significant results are highlighted in the Table 2. Moreover, a multiple linear regression analysis was performed to assess the contribution of each variable to the variation of the LuxR frequencies among the studied organisms (Table S2).

**Table 2:** Correlations between the different LuxR sub-families and genetic/ecological variables - the statistical significant correlations are highlighted in bold.

	GenSize (N = 53)	%G+C (N = 53)	Temp (N = 53)	Mot (N = 52)	OxyReq (N = 53)	Spor (N = 51)	CellArr (N = 45)	HostDep (N = 53)	HostKind (N = 29)	T,PK&NRP (N = 53)	other SM (N = 53)
REC+LuxR	**r_s_ = 0.534**	**r_s_ = 0.554**	r_s_ = 0.086	r_s_ = −0.134	r_s_ = 0.254	**r_s_ = 0.513**	**r_s_ = 0.761**	**r_s_ = **−**0.448**	**r_s_ = **−**0.522**	**r_s_ = 0.452**	r_s_ = 0.229
	p = 0.000	p = 0.000	p = 0.539	p = 0.344	p = 0.067	p = 0.000	p = 0.000	p = 0.001	p = 0.004	p = 0.001	p = 0.100
	β = 0.989	β = 0.994	β = 0.095	β = 0.158	β = 0.456	β = 0.977	β = 1.000	β = 0.931	β = 0.852	β = 0.936	β = 0.384
	**η = 0.849**	**η = 0.615**	η = 0.013	η = 0.118	η = 0.255	**η = 0.587**	**η = 0.752**	**η = 0.415**	**η = 0.446**	**η = 0.750**	η = 0.595
LuxR	**r_s_ = 0.804**	**r_s_ = 0.497**	r_s_ = −0.218	r_s_ = 0.226	**r_s_ = 0.452**	**r_s_ = 0.445**	**r_s_ = 0.588**	**r_s_ = **−**0.628**	**r_s_ = **−**0.554**	**r_s_ = 0.618**	**r_s_ = 0.377**
	p = 0.000	p = 0.000	p = 0.117	p = 0.107	p = 0.001	p = 0.001	p = 0,000	p = 0.000	p = 0.002	p = 0.000	p = 0.005
	β = 1.00	β = 0.973	β = 0.353	β = 0.370	β = 0.936	β = 0.917	β = 0.993	β = 1.000	β = 0.900	β = 0.999	β = 0.808
	**η = 0.864**	**η = 0.473**	η = 0.191	η = 0.165	**η = 0.400**	**η = 0.446**	**η = 0.702**	**η = 0.566**	**η = 0.584**	**η = 0.730**	**η = 0.619**
other	**r_s_ = 0.305**	r_s_ = 0.020	r_s_ = −0.048	r_s_ = −0.065	r_s_ = 0.101	r_s_ = −0.112	**r_s_ = 0.302**	r_s_ = −0.210	–	r_s_ = 0.232	r_s_ = 0.264
	p = 0.026	p = 0.885	p = 0.730	p = 0.646	p = 0.473	p = 0.434	p = 0.044	p = 0.130	–	p = 0.095	p = 0.056
	β = 0.615	β = 0.052	β = 0.064	β = 0.075	β = 0.111	β = 0.123	β = 0.534	β = 0.332	–	β = 0.393	β = 0.488
	**η = 0.551**	η = 0.209	η = 0.043	η = 0.063	η = 0.090	η = 0.106	**η = 0.326**	η = 0.202	η = 0.202	η = 0.494	η = 0.400
total n° of sequences	**r_s_ = 0.788**	**r_s_ = 0.627**	r_s_ = −0.033	r_s_ = 0.146	**r_s_ = 0.474**	**r_s_ = 0.472**	**r_s_ = 0.697**	**r_s_ = **−**0.627**	**r_s_ = **−**0.570**	**r_s_ = 0.614**	**r_s_ = 0.425**
	p = 0.000	p = 0.000	p = 0.817	p = 0.303	p = 0.000	p = 0.000	p = 0.000	p = 0.000	p = 0.001	p = 0.000	p = 0.002
	β = 1.00	β = 1.000	β = 0.056	β = 0.180	β = 0.957	β = 0.948	β = 1.000	β = 1.000	β = 0.920	β = 0.999	β = 0.900
	**η = 0.926**	**η = 0.557**	η = 0.088	η = 0.093	**η = 0.373**	**η = 0.498**	**η = 0.769**	**η = 0.536**	**η = 0.551**	**η = 0.764**	**η = 0.704**

GenSize, Genome size; %G+C, percentage of GC in the genome; Temp, Temperature; OxyReq, oxygen requirement; CellArr, cellular arrangement; HostDep, host dependency; HostKind, host kind; T, PK&NRP, terpenoids, polyketides and non-ribossomal protein synthase machinery; SM, secondary metabolism; r_s_, Spearman Rank Order Correlations; β, Power (Refined Fischer Z); η, Eta coefficient.

The “other” LuxR sub-families, i.e., those besides REC+LuxR and LuxR, are only correlated with the genome size and the cellular arrangement (with a borderline p-value of 0.044). This is probably due to their small number and their analysis as a whole (different subfamilies could present different correlations that ended up masked by the general tendency).

Regarding the genome size, the tests indicate a positive correlation with both the total number of sequences and each of the subfamilies. This result is in accordance with previous works in which it was demonstrated that the number of HTH regulators increases as the genome increase, since a higher number of genes implies a greater complexity in the regulatory systems [22]. Moreover, the genomes composition in terms of %G+C is also positively correlated with the frequency of the two main groups of LuxR proteins and the total number of sequences. However, the results from the multiple regression analysis (Table S2) show that the effect of this variable is not significant when controlling for other variables, and therefore this aspect will not be further explored in this manuscript.

Concerning the optimal growing temperature and the organisms motility, no significant correlations were found, suggesting that LuxR family regulators are not significantly involved in these aspects of actinobacterial organisms. QS, and particularly LuxR systems, are known to control the switch from swimming or sliding to swarming in several well studied Gram-negative models, such as *Serratia* spp. and *Pseudomonas aeruginosa* [23], [24]. However, the absence of these motility patterns in Actinobacteria justifies the lack of correlations observed.

The oxygen requirement is positively correlated with the occurrence of single domain LuxR-containing sequences (Table 2). Although this correlation is not particularly strong (r_s_ = 0.452), the multiple regression analysis shows it has a significant effect on the variance of the total number of LuxR sequences (Table S2). This suggests that LuxR regulators may have a role in the overall aerobic metabolism and/or in the response to oxidative stress. The existence of a conspicuous relationship between oxidative stress tolerance and the ability to communicate through quorum-sensing mechanisms has already been demonstrated both in Gram-negative and in Gram-positive organisms [25]–[27]. Moreover, the fact that only this particular sub-family, which lacks a signal recognition domain, is correlated with the oxygen requirement is in agreement with what is known for other redox-dependent regulators functionality. In fact, O_2_ and its reduced species can easily enter the cells without a specialized transporter, and the activation of the redox-dependent regulators is achieved not through a typical signal-binding process, but rather by a redox-induced structural modification (see, for instance, [28]). Consequently, the LuxR proteins responding (and therefore correlated) to the oxygen presence are likely activated by similar structural-changing processes, and do not require a signal transduction domain.

Sporulation and cellular arrangement are also positively correlated with both REC+LuxR and single domain LuxR sub-families, as well as with the totality of LuxR-type proteins (Table 2). These results indicate that the morphology of the actinobacterial organisms is at least partially regulated by LuxR regulators. In fact, the higher the complexity of cellular arrangement or the probability of being a sporulating organism, the higher the tendency to positively select LuxR regulators and therefore the higher their frequency.

The kind of relationship with a given host and the nature of that host are two other factors to be considered in the distribution of LuxR proteins in Actinobacteria. In fact, the frequency of LuxR proteins is negatively correlated (r_s_ = −0.627) with the dependency towards a certain host - meaning that the higher the dependence, the smaller the number of LuxR regulators - and with the presence of animals as hosts (r_s_ = −0.570) (Table 2). It should be noted, however, that obligate parasites or symbionts (the organisms that present the highest dependence towards a given host according to our classification system -Table S1) are known to suffer a process of gene deletion, having consequently smaller genomes [29]. And in fact, taking into account the effect of the genome size, the host dependency is no longer a significant factor in the distribution of the LuxR regulators (Table S2). On the other hand, the observed positive correlation between plants as a host and the number of LuxR-containing sequences is significant and it has been previously reported [30]. One can hypothesize that the absence of a circulating acquired immune system in plants reliefs the selective pressure on the associated bacteria, favoring more complex and fine-tuned regulated microbial populations. The secretion of cell-to-cell signaling molecules, to which LuxR may respond, is likely facilitated in plant-associated organisms when compared to the animal-associated ones, justifying the selection of a greater number of homologs.

Finally, the distribution of LuxR regulators was show to be positively correlated with the potential to engage into secondary metabolic pathways related with the metabolism of terpenoids, polyketides (PK), non-ribosomal peptides (NRP) or others (Table 2). The multiple regression analysis shows that this effect is not significant with a borderline p value of 0.08 (Table S2), which is probably related with the fact that secondary metabolism is usually found in organisms with a large genome and a more diversified genetic machinery. Consequently, the effect of the variable concerning secondary metabolism is no longer significant when the genome size is taken into account. Nevertheless, this topic deserves some discussion, given the importance of secondary metabolism among Actinobacteria and the key role played by the associated LuxR regulators. Indeed, the most conspicuous lineage-specific expansions that this family of regulators suffered was in *Streptomyces* spp., a group of organisms well-known for their ability to produce a wide range of bioactive secondary metabolites. Adding to this, *Salinispora* spp. also have a considerably high number of LuxR regulators when compared with other organisms with identical genome sizes. Previously published studies demonstrate a major role of LuxR regulators in the PK biosynthesis in *Streptomyces* spp., either as pathway-specific [31], [32] or as pleiotropic regulators [14]. The importance of these LuxR transcription factors can actually extend to the biotechnology field, since it has been shown that the induced expression of a LuxR regulator triggered the translation of a large cryptic biosynthetic cluster in *Streptomyces ambofaciens* [33]. Moreover, the overexpression of *salR2*, a *luxR* homolog of *Salinispora tropica*, was shown to enhance the production of salinosporamide A through the activation of the biosynthesis of its specific precursor chloroethylmalonyl-CoA [34].

With the exception of the correlation with oxygen requirement and the production of secondary metabolites other than terpenoids, PKs and NRPs, all the other correlations established by the two main sub-families of the LuxR proteins (LuxR and REC+LuxR) are essentially the same (Table 2). This suggests that the selective pressures exerted by the ecological niche *per se* were not the main evolutionary force driving the appearance of an extra domain fused to the LuxR, in which case the correlations of LuxR and REC+LuxR with the different ecological variables would likely vary. In fact, the presence of a signal-recognition domain coupled to LuxR is likely the result of an overall need to physically uncouple the signal sensing from the response regulation, and not the result of specific ecologically-related selective pressures.

### *In silico* Annotation of the Family of LuxR Proteins

One of the striking characteristics of the LuxR-regulators is their involvement in a wide range of molecular functions and cellular processes [10]. To have an overview of the main roles played by the LuxR regulators in Actinobacteria, a functional annotation based on the GO hierarchical system was carried out, and the GO terms assignment between the different ecological categories considered was compared. The results reveal that there are several GO terms which assignment is statistically different between mutually exclusive ecological classification terms, suggesting that the LuxR-containing sequences have become specialized in different functions according to the ecological niche to which the organisms have adapted (Fig. S3). So, opposite to the physical way by which this regulation is exerted (TCS vs single LuxR domain transcription factors), the functions modulated by these regulators are indeed dependent on ecological variables (Fig. S3). This also explains the observed extensive duplication of LuxR-containing sub-families in specific species/genus. Gene duplication is known to be a major route for genomes evolution. However, unless duplicated genes evolve and give rise to paralogous proteins that, while maintaining the same general function, differ in specific functional details, they tend to be eliminated from the genomes [21]. As it has been shown for other transcriptional regulators, it seems that LuxR sub-families have suffered several duplications followed by functional specialization, and the resulting genes were either lost or fixed in the organisms according to the selective pressures exerted by their ecological context. This differential loss/fixation originated the variable pattern of functionalities attributed to the LuxR sequences from organisms with different environmental backgrounds (Fig. S3).

### LuxR and Bacterial Virulence: the *Mycobacterium* spp. Particular Case

**Figure 4:**
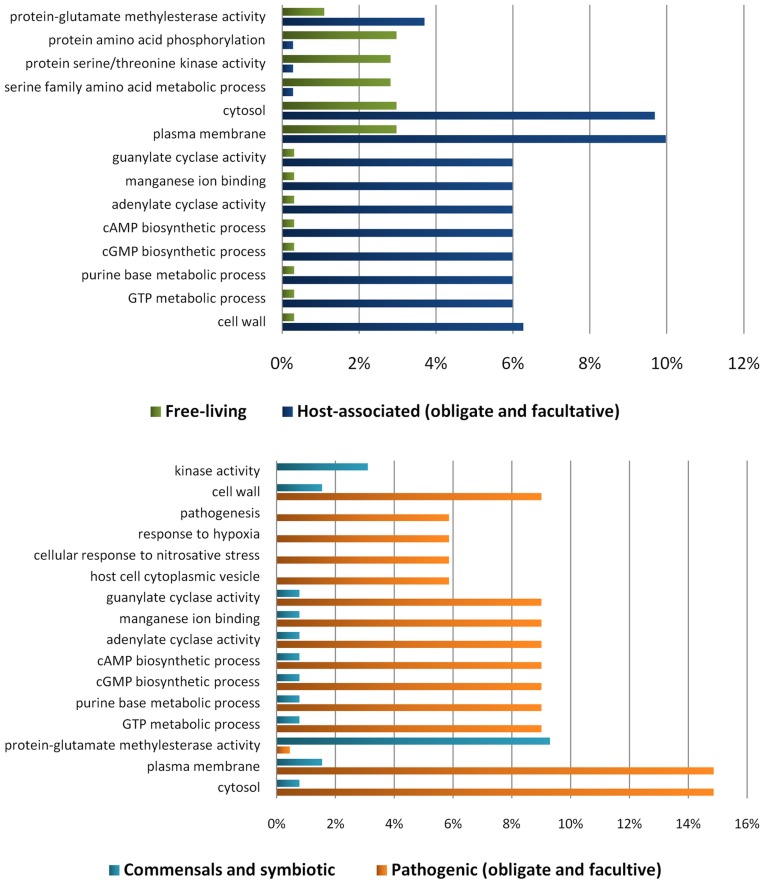
Significant differences in the GO terms-based functional annotation of the LuxR regulators belonging to specific categories. The GO terms shown in the graphs correspond to the most specific ones among those that were considered significant (i.e., with a p-value by False Discovery Rate control below 0.05).

In previously studied Gram-negative bacteria, LuxR regulators (particularly those involved in QS systems) are known to be crucial factors in the virulence of pathogenic organisms [35]–[37]. To determine if that was also the case with actinobacterial LuxR sequences, we analyzed whether there was any significant difference in the GO terms assignment when comparing the LuxR proteins classification of host-associated and free-living organisms, as well as of pathogenic organisms and other host-associated organisms (Fig. 4). LuxR sequences from host-associated organisms are statistically associated with cyclic nucleotides and nucleotides metabolism in general, whereas LuxR sequences from free-living organisms are apparently more engaged into protein metabolism (Fig. 4A). Adding to this, virulence-related terms such as pathogenesis, response to hypoxia, cellular response to nitrosative stress and host cell cytoplasmic vesicle are represented among the LuxR proteins from pathogenic organisms and not in those from other host-associated bacteria (Fig. 4B). These results suggest that the LuxR-family of proteins is important in Actinobacteria virulence.

In this context, it is important to highlight the particular case of the CHD+LuxR sub-family. Cyclase homology domains (CHD) are often found coupled with several different regulatory modules, granting them the ability to sense a large variety of input signals besides their role as intracellular cAMP generators [38]. As it was said in the phylogeny section, the presence of CHD+LuxR sub-family in Actinobacteria is restricted to members of the *Mycobacterium* genus (Fig. 3). Moreover, and within *Mycobacterium* species, CHD+LuxR proteins occur in 5 (out of the 11 analyzed) pathogenic *Mycobacterium* species, including all members of the *M. tuberculosis* complex, and only in 1 (out of the 5 analyzed) non-pathogenic *Mycobacterium* species, suggesting a role of CHD+LuxR proteins in virulence (Table 1 and Fig. 3). Interestingly, these sub-family proteins from *M. tuberculosis* have been mentioned in previously published studies, although none of these studies was specifically focused on these proteins or in their putative QS facets. The gene coding for one of the proteins, Rv2488c, was found to be 59-fold upregulated in a putative metalloendopeptidase (Rv0918c) mutant with an hypervirulent phenotype in mouse models [39]. That same gene was 2-fold downregulated in a mutant strain lacking the 2-component system *senX3*-*regX3*, which had an intramacrophage growth defect and attenuated virulence phenotype [40]. Another of these proteins, encoded by Rv0386, was shown to have an alternative substrate-binding mechanism regarding its cyclase activity [41], and infection with *M. tuberculosis* that do not express Rv0386 resulted in a decreased bacterial-derived intramacrophage cAMP, tumour necrosis factor- (TNF) production and bacterial survival [42]. Finally, a *M. tuberculosis* strain overexpressing an alternative sigma factor, SigF, whose absence causes a partially attenuated phenotype, was found to have a 14-fold increase in the anti-sense mRNA transcript of Rv1358, another CHD+LuxR encoding gene [43].These studies further support the association between these regulators and virulence in mycobacteria, while raising the question of the role played by the LuxR domain in these proteins and suggesting cAMP or cGMP as potential signals. It should be stressed that, as in almost all Actinobacteria, the knowledge on QS in *Mycobacterium* genus is scarce and limited to indirect evidences, such as the induction of biofilm formation in *M. avium* after exposure to AI-2 [44] and the QS-like expression of the tissue-damage related transcriptional regulator WhiB3 in *M. tuberculosis* [45]. However, an association between a LuxR regulator (MAP0482) and *M. avium* virulence and adaptation to the host has recently been published [46], further reinforcing the hypothesis that LuxR regulators are important in the pathogenicity of mycobacterial infections, either through QS or other signaling pathways. This knowledge has key promising applications in the biomedicine field, since the elucidation of the LuxR-regulated pathways can lead to the identification of new drug targets aimed at virulence inhibition or even new diagnostics methods based on the bacterial release of specific virulence-related QS signals.

### Conclusions and Future Directions

The present study reveals a great diversity of the LuxR family of proteins in Actinobacteria. Although these regulators are paradigmatic transcription factors in the QS of well-studied Gram-negative models, they have been seldom described in the Gram-positive organisms. To the best of our knowledge, the present report is the first broad phylogenomic approach of these regulators in Actinobacteria, using a multidimensional perspective to understand their distribution, phylogenetic history and functionality.

There are two main groups of LuxR regulators in Actinobacteria: one that carries a single LuxR domain and that appears to be a transcription factor; and another one that, in addition to LuxR, carries an extra domain related with signal recognition/transduction, which resembles the TCS RR architecture. The evolution of these two groups occurred through a series of gene fusion/fission and duplication events, punctually marked by HGT and gene loss. According to our results and to the variables accounted for, the LuxR fusion with other domains is not the result of any specific ecological selective pressure, but rather by an overall need to uncouple the signal sensor from the response regulation. The ecological variables addressed have, however, shaped the functionality of the LuxR regulators in general. Particularly in the case of pathogenic organisms, LuxR regulators appear to play a role in the modulation of virulence. This might be of particular importance in the *Mycobacterium* genus, in which an almost exclusive group of LuxR regulators seems to be implied in virulence. Therefore, LuxR regulators appear here as potential targets to be explored in the fight against actinobacterial infections, namely those caused by mycobacteria. We believe that this study, by exploring all the possible LuxR regulators, their evolutionary history, their functionality and exposing their possible redundancy, offers a well-established theoretical background for future biomedical approaches.

## Materials and Methods

### Domain Search and Sequences Retrieval

Initially, all the LuxR family regulators (i.e., proteins with a LuxR regulatory domain) were retrieved from a set of Actinobacteria species which proteome was fully available both in Pfam 24.0 platform (based on UniProtKB version 15.6) and in the National Center for Biotechnology Information (NCBI). These species were chosen in order to be representative of the entire phylum in terms of phylogeny, morphology, ecological niche and metabolism. Given the typical lack of conservation and the short size of these transcriptions factors, a domain-based approach using two different filters was employed. Initially, Pfam 24.0 platform [47] was used to identify all proteins present in the selected Actinobacteria that have at least one GerE domain (Pfam-A entry PF00196). The GerE HMM profile is composed of 58 residues and corresponds to the HTH C-terminal of the LuxR proteins family. Following this, and since different methods used to search for specific protein domains commonly give rise to different matches [48], the obtained set of sequences was optimized by filtering it with the CD-search from NCBI using the Conserved Domains Database (CDD) v3.05 [49]. The NCBI CDD imports conserved domains from outside sources and refines them using 3D structural information. The curated domain that corresponds to LuxR is named LuxR_C_like (cd06170), and is precisely based on that from Pfam, therefore spanning the C-terminal of the LuxR proteins family. All sequences previously retrieved from Pfam were scanned with CD-search (CDD v3.05), and only those that had LuxR_C_like as a specific hit (superfamily and multidomains hits without cd06170 were rejected) were retained for further analysis. Additional domains were considered whenever a specific hit (superfamily and multidomains hits were rejected) besides LuxR was identified in the CD-search.

### Multiple Alignments and Phylogenetic Trees

Protein sequences (containing the LuxR domain) and DNA sequences (of the species 16S rRNA subunit) were aligned using the ClustalW algorithm present in the Geneious Pro 5.1.7 package [50]. The 16S alignment was used to build a Neighbor-Joining (NJ) unrooted phylogenetic tree with the Geneious Tree Builder, having Jukes-Cantor (JC) [51] as the evolutionary model and 10000 replicates for the bootstrap analyses. The same alignment was used to compute a Maximum-Likelihood (ML) tree using the PhyML, available in the Geneious Pro Package [50], [52], also using the JC model with a Gamma-distributed rate heterogeneity with 4 substitution rates, a transition/transversion ratio of 4 and 100 replicates for the bootstrap analyses. The LuxR-containing sequences alignment was used to construct both NJ and ML unrooted phylogenetic trees using the Geneious Tree Builder and the PhyML, respectively [50], [52]. The models used were the JC for the NJ tree, and the WAG [53] for the ML tree, whereas statistical support was computed by bootstrap analysis in the NJ tree (using 1000 pseudo-replicates) and by the SH-like interpretation of the aLRT (approximate likelihood ratio test) in the ML tree.

### Statistical Analysis

The Spearman Rank Order Correlations and the multiple regression analyses were computed using STATISTICA10 (StatSoft). The Eta coefficients were computed using IBM SPSS Statistics 19 (IBM). Data for the classification of each organism was retrieved from NCBI, IMG (Integrated Microbial Genomes from Doe Joint Genome Institute) and KEGG (Kyoto Encyclopedia of Genes and Genomes). T,PK&NRP and other secondary metabolism variables were defined as the number of genetic pathways each organism had assigned in KEGG under the categories “1.9 Metabolism of Terpenoids and Polyketides”, and “1.10 Biosynthesis of Other Secondary Metabolites”, respectively, and despite the completeness of the pathway. To calculate Spearman Rank Order Correlations, the variables considered were transformed into ordinal variables according to the coding system available in Table S1, with exception of the variables related to secondary metabolism (T, PK & NRP and other SM), in which the original metric variables were used. To calculate the Eta coefficient, the dependent variables considered were the frequencies of the different LuxR subfamilies in their ordinal scales, and the independent variables were the categorical classifications of each studied characteristic, with the exception of GenSize and %G+C, for which the ordinal scales were used, and T,PK&NRP and other SM, for which the original metric scales were used.

### *In silico* Functional Annotation

The *in silico* functional annotation was carried out using the Blast2GO V.2.5.0 suite [54]. Initial blasting step was performed using the blastP program (Sept 2011), in the Q-blast-NCBI mode, against the nr (non-redundant database) and using the default parameters (HSP cutoff of 33 and retrieval of 50 blast hits with a blast expected value lower that 1×10^−3^), recommended for sequences with similarities mainly above 65% [54], a condition fulfilled in this work. The mapping and annotation steps were done according to default parameters. Additional Interpro scan and Annex steps were run to enrich and optimize the annotation. The statistical assessment of the differential distribution of Gene Ontology (GO) terms between different ecological categories was also carried out in the Blast2GO suite, performing an enrichment analysis that uses the Fischer’s Exact Test and corrects for multiple testing. The sequences of defined groups were compared to those of the reference group, and the GO terms were filtered by the FDR (p-value by False Discovery Rate control) of 0.05. To facilitate the interpretation and avoid redundancy, only the most specific GO terms are shown in the graphs.

## Supporting Information

Figure S1 **Maximum-likelihood tree of the species considered in this study.** Numbers beside nodes indicate boostrap support.
(PDF)

Figure S2 **Maximum-likelihood tree of all LuxR-containing sequences retrieved from Actinobacteria.** Sequences are indicated by their locus tag followed by a letter code corresponding to their domain composition (L, LuxR; RL, REC+LuxR; CL, CHD+LuxR; HL, HDc+LuxR; AL, AAA+LuxR; PL, Pkc+LuxR; FL, FHA+LuxR; TL, TRP+LuxR; LC, LuxR+ CSP_CDS; PsL, PAS+LuxR; PTL, Pkc+ TRP+LuxR). A colour-code is used to distinguish between the two main subfamilies: single-domain LuxR proteins are in green, whereas REC+LuxR proteins are in red. Numbers in the branches correspond to the SH-like interpretation of the aLRT values.
(PDF)

Figure S3 **Significant differences in the GO terms-based functional annotation of the LuxR regulators from different ecological categories.**
(PDF)

Table S1 **Classification of the considered actinobacterial organisms regarding a series of genetic and ecological variables.**
(PDF)

Table S2 **Multiple regression analysis of the variables considered in this study regarding the distribution of the total number of LuxR regulators among the actinobacteria considered.**
(PDF)
