# Supplementary figures and images for: A Walk into the LuxR Regulators of Actinobacteria: Phylogenomic Distribution and Functional Diversity
Source: PLoS One. 2012 Oct 8;7(10):e46758. doi: 10.1371/journal.pone.0046758 (PMC3466318; doi:10.1371/journal.pone.0046758)

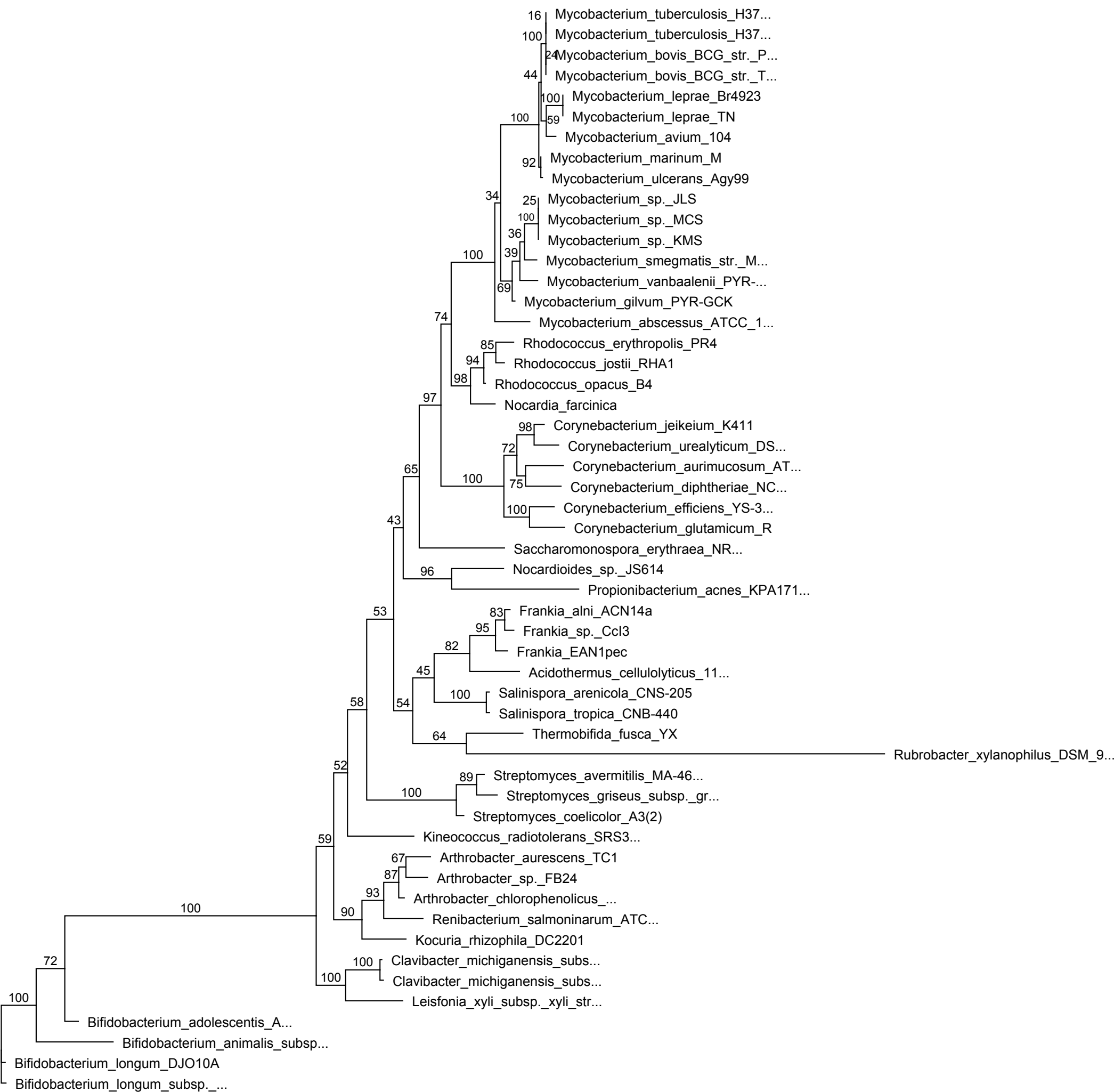

0.3

Supplement: Figure S1 — Maximum-likelihood tree of the species considered in this study. Numbers beside nodes indicate boostrap support. (PDF) [file pone.0046758.s001.pdf]
