# Supplementary material for: A Walk into the LuxR Regulators of Actinobacteria: Phylogenomic Distribution and Functional Diversity
Source: PLoS One. 2012 Oct 8;7(10):e46758. doi: 10.1371/journal.pone.0046758 (PMC3466318; doi:10.1371/journal.pone.0046758)
Supplement: Figure S3 — Significant differences in the GO terms-based functional annotation of the LuxR regulators from different ecological categories. (PDF) [file pone.0046758.s003.pdf]

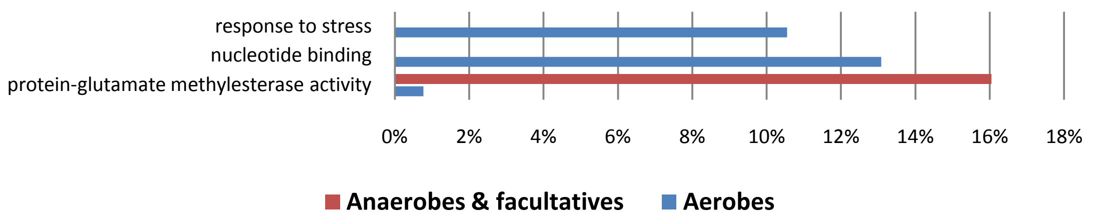
response to stress
nucleotide binding
protein-glutamate methylesterase activity
0%
2%
4%
6%
8%
10%
12%
14%
16%
18%
Anaerobes & facultatives
Aerobes

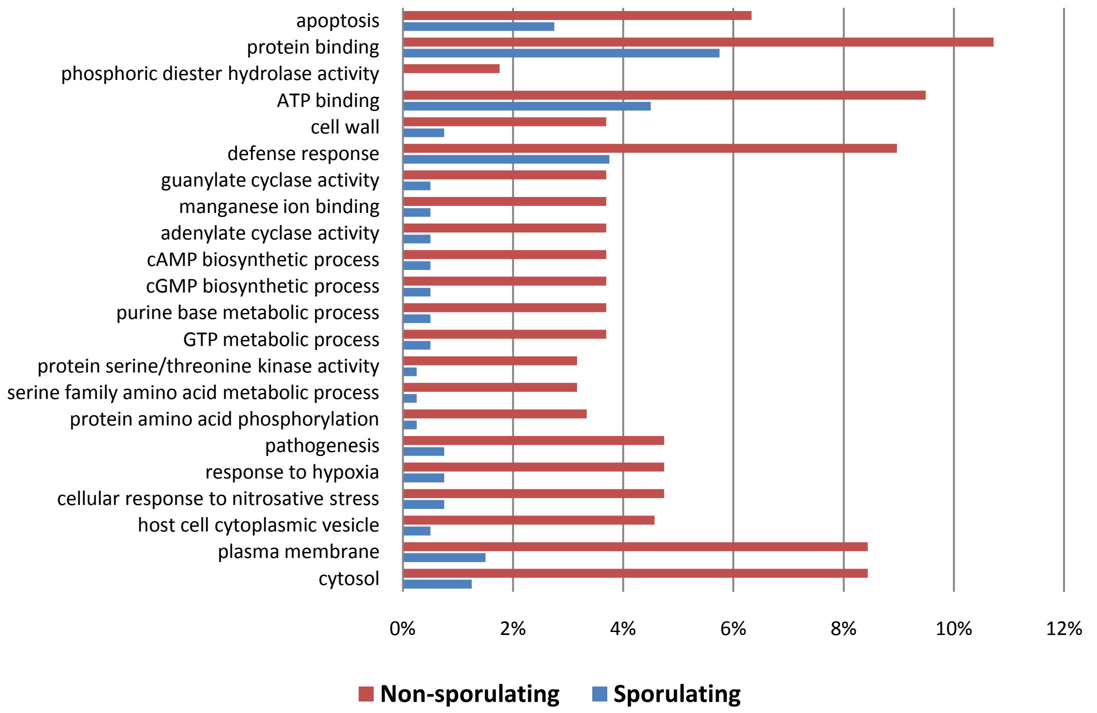
apoptosis
protein binding
phosphoric diester hydrolase activity
ATP binding
cell wall
defense response
guanylate cyclase activity
manganese ion binding
adenylate cyclase activity
cAMP biosynthetic process
cGMP biosynthetic process
purine base metabolic process
GTP metabolic process
protein serine/threonine kinase activity
serine family amino acid metabolic process
protein amino acid phosphorylation
pathogenesis
response to hypoxia
cellular response to nitrosative stress
host cell cytoplasmic vesicle
plasma membrane
cytosol
0%
2%
4%
6%
8%
10%
12%
Non-sporulating
Sporulating

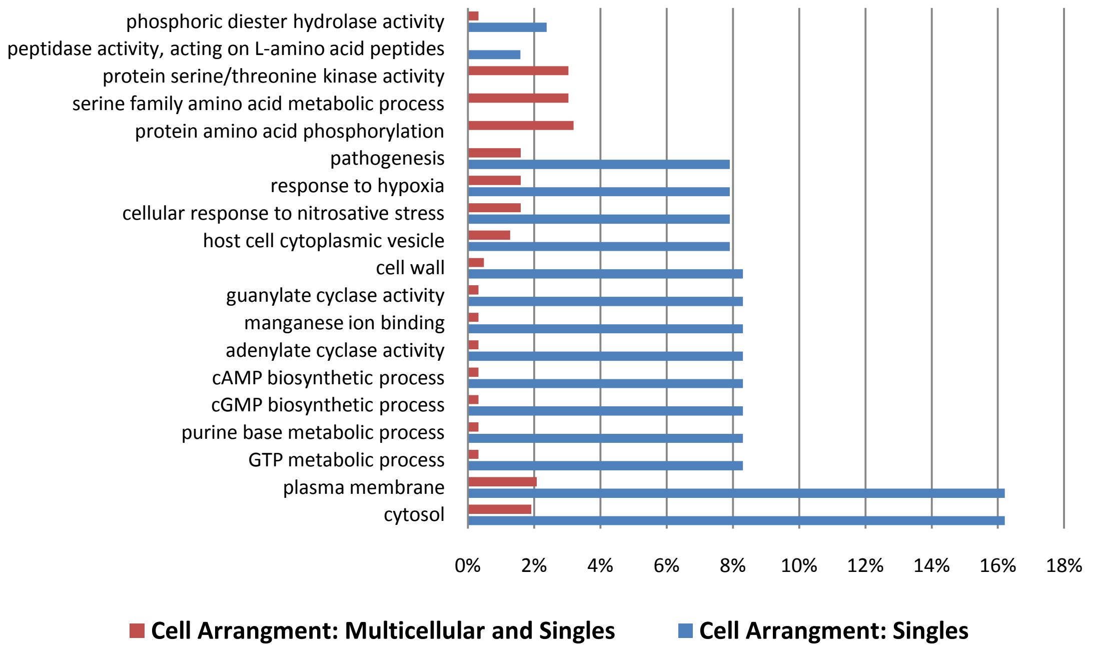
phosphoric diester hydrolase activity
peptidase activity, acting on L-amino acid peptides
protein serine/threonine kinase activity
serine family amino acid metabolic process
protein amino acid phosphorylation
pathogenesis
response to hypoxia
cellular response to nitrosative stress
host cell cytoplasmic vesicle
cell wall
guanylate cyclase activity
manganese ion binding
adenylate cyclase activity
cAMP biosynthetic process
cGMP biosynthetic process
purine base metabolic process
GTP metabolic process
plasma membrane
cytosol
0%
2%
4%
6%
8%
10%
12%
14%
16%
18%
Cell Arrangment: Multicellular and Singles
Cell Arrangment: Singles

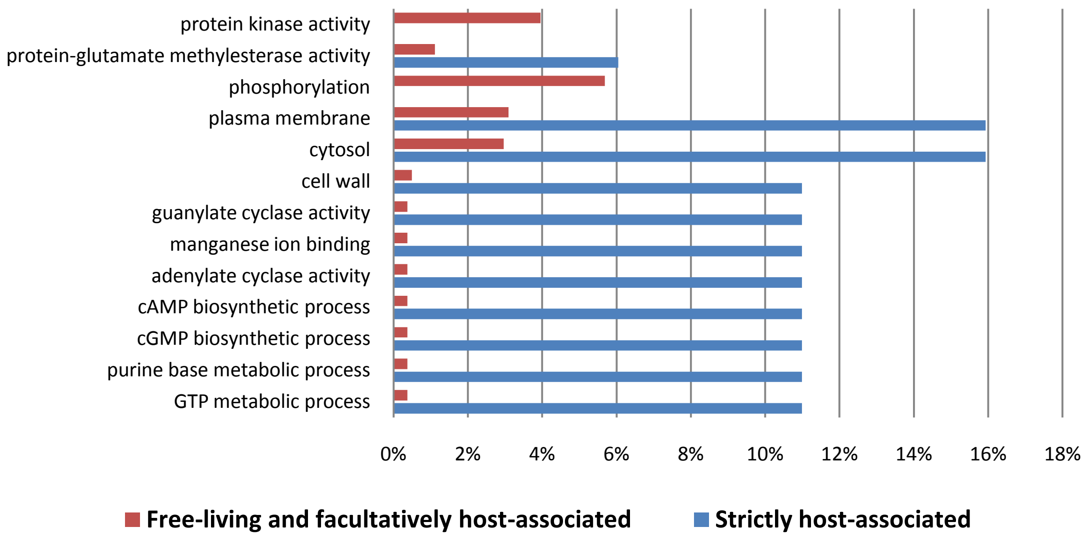
protein kinase activity
protein-glutamate methylesterase activity
phosphorylation
plasma membrane
cytosol
cell wall
guanylate cyclase activity
manganese ion binding
adenylate cyclase activity
cAMP biosynthetic process
cGMP biosynthetic process
purine base metabolic process
GTP metabolic process
0%
2%
4%
6%
8%
10%
12%
14%
16%
18%
Free-living and facultatively host-associated
Strictly host-associated

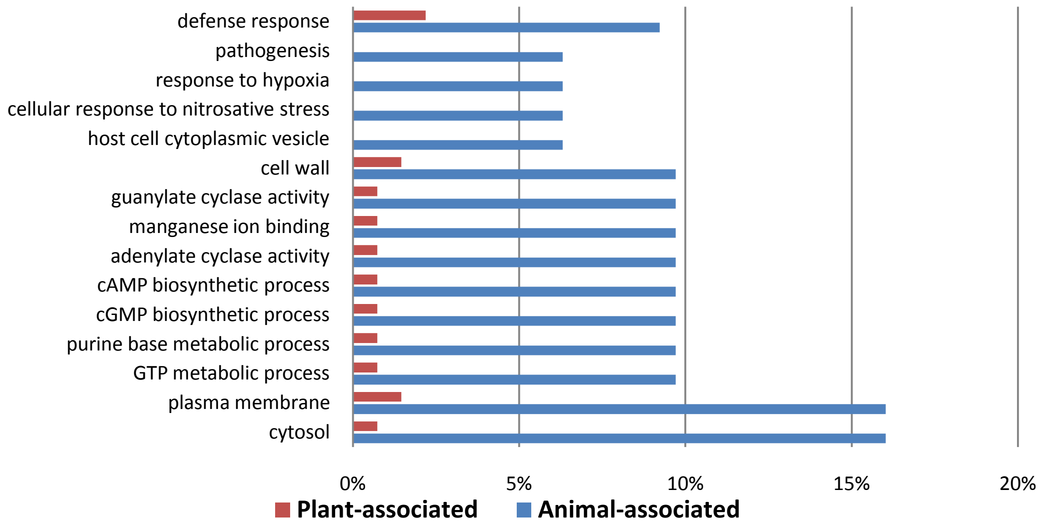
defense response
pathogenesis
response to hypoxia
cellular response to nitrosative stress
host cell cytoplasmic vesicle
cell wall
guanylate cyclase activity
manganese ion binding
adenylate cyclase activity
cAMP biosynthetic process
cGMP biosynthetic process
purine base metabolic process
GTP metabolic process
plasma membrane
cytosol
0%
5%
10%
15%
20%
Plant-associated
Animal-associated

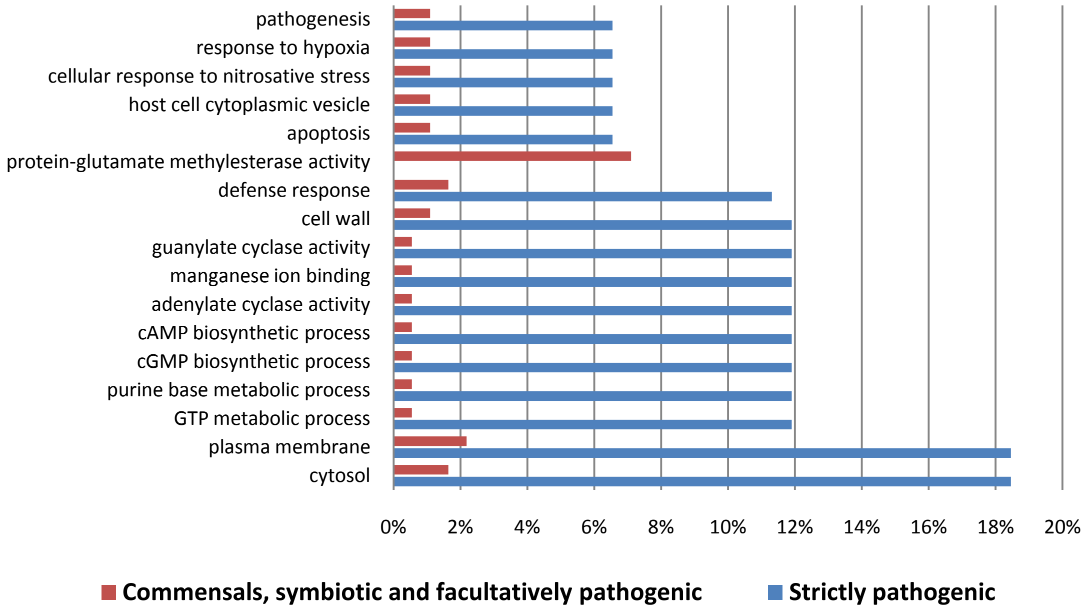
pathogenesis
response to hypoxia
cellular response to nitrosative stress
host cell cytoplasmic vesicle
apoptosis
protein-glutamate methylesterase activity
defense response
cell wall
guanylate cyclase activity
manganese ion binding
adenylate cyclase activity
cAMP biosynthetic process
cGMP biosynthetic process
purine base metabolic process
GTP metabolic process
plasma membrane
cytosol
0%
2%
4%
6%
8%
10%
12%
14%
16%
18%
20%
Commensals, symbiotic and facultatively pathogenic
Strictly pathogenic
