# Supplementary material for: A Walk into the LuxR Regulators of Actinobacteria: Phylogenomic Distribution and Functional Diversity
Source: PLoS One. 2012 Oct 8;7(10):e46758. doi: 10.1371/journal.pone.0046758 (PMC3466318; doi:10.1371/journal.pone.0046758)
Supplement: Table S1 — Classification of the considered actinobacterial organisms regarding a series of genetic and ecological variables. (PDF) [file pone.0046758.s004.pdf]

Species name	#REC+LuxR	#LuxR	#other	#SeqTot	GenSize	%G+C	TºC	Mot	OxyReq	Spor	CellArr	Hab	HostDep	Host	BioRel	#T+PK+NRP	#other sec met	#total sec met
Acidothermus cellulolyticus 11B	6	0	0	6	2.4	66.9	t		ae	y		W	N	N	N	5	2	7
Arthrobacter aurescens TC1	5	11	0	16	5.2	62.4	m	y	ae	n		S	N	N	N	5	3	8
Arthrobacter chlorophenolicus A6	5	11	1	17	5.0	66.0	m	y	ae	n		S	N	N	N	5	3	8
Arthrobacter sp. FB24	5	9	0	14	5.1	65.4	m	y	ae	n		S	N	N	N	4	2	6
Bifidobacterium adolescentis ATCC15703	7	0	0	7	2.1	59.2	m	n	an	n	S	H	O	A	F	3	2	5
Bifidobacterium animalis subsp. *lactis* AD011	5	0	0	5	1.9	60.5	m	n	an	n		H	O	A	F	2	2	4
Bifidobacterium longum DJO10A	4	0	0	4	2.4	60.1	m	n	an	n	SP	H	O	A	F	2	2	4
Bifidobacterium longum subsp. *infantis* ATCC15697	14	0	0	14	2.8	59.9	m	n	an	n	SP	H	O	A	F	1	2	3
Clavibacter michiganensis subsp. *michiganensis* NCPPB 382	13	4	0	17	3.4	72.5	m	n	ae	n	SP	H	O	P	P	3	2	5
Clavibacter michiganensis subsp. *sepedonicus*	10	5	0	15	3.4	72.4	m	n	ae	n	SP	H	O	P	P	3	3	6
Corynebacterium aurimucosum ATCC 700975	7	1	0	8	2.8	60.5	m	n	f	n	S	H	O	A	F/P	3	2	5
Corynebacterium diphtheriae NCTC 13129	5	1	0	6	2.5	53.5	m	n	f	n	S	H	O	A	P	4	2	6
Corynebacterium efficiens YS-314	6	4	0	10	3.2	62.9	m	n	f	n	S	S	N	N	N	4	2	6
Corynebacterium glutamicum R	5	3	0	8	3.4	54.1	m	n	f	n	S	M	N	N	N	4	2	6
Corynebacterium jeikeium K411	3	1	0	4	2.5	61.4	m	n	f	n	S	H	O	A	F/P	5	2	7
Corynebacterium urealyticum DSM 7109	3	1	0	4	2.4	64.2	m	n	ae	n	S	H	O	A	F/P	5	2	7
Frankia alni ACN14a	13	12	0	25	7.5	72.8	m	n	ae	y	P	M	F	P	S	10	2	12
Frankia EAN1pec	31	16	1	48	9.0	71.2	m	n	ae	y	P	M	F	P	S	10	2	12
Frankia sp. CcI3	6	12	0	18	5.4	70.1	m	n	ae	y	P	M	F	P	S	10	2	12
Kineococcus radiotolerans SRS30216	18	10	0	28	5.0	74.2	m	y	ae	n	SP	M	N	N	N	4	3	7
Kocuria rhizophila DC2201	7	1	0	8	2.7	71.2	m	n	ae		SP	M	F			4	2	6
Leisfonia xyli subsp. *xyli* str. CTCB07	7	7	0	14	2.6	67.7	m	n	ae		SP	H	O	P	P	3	2	5
Mycobacterium abscessus ATCC 19977	6	3	0	9	5.1	64.1	m	n	ae	n	S	M	F	A	P	6	3	9
Mycobacterium avium 104	4	2	2	8	5.5	69.0	m	n	ae	n	S	M	F	A	P	6	3	9
Mycobacterium bovis BCG str. Pasteur 1173P2	2	2	3	7	4.4	65.6	m	n	ae	n	S	H	O	A	P	6	3	9
Mycobacterium bovis BCG str. Tokyo 172	2	2	3	7	4.4	65.6	m	n	ae	n	S	H	O	A	P	6	3	9
Mycobacterium gilvum PYR-GCK	6	8	1	15	6.0	67.7	m	n	ae	n	S	S	N	N	N	6	2	8
Mycobacterium leprae Br4923	0	1	0	1	3.3	57.8	m	n	ae	n	S	H	O	A	P	4	2	6
Mycobacterium leprae TN	0	1	0	1	3.3	57.8	m	n	ae	n	S	H	O	A	P	4	2	6
Mycobacterium marinum M	5	4	2	11	6.7	65.7	m	n	ae	n	S	H	O	A	P	6	3	9
Mycobacterium smegmatis str. MC2 155	13	18	1	32	7.0	67.4	m	n	ae	n	S	H	O	A	F/P	6	4	10
Mycobacterium sp. JLS	7	12	2	21	6.1	68.4	m	n	ae	n	S	S	N	N	N	6	2	8
Mycobacterium sp. KMS	7	10	1	18	6.3	68.2	m	n	ae	n	S	S	N	N	N	6	2	8
Mycobacterium sp. MCS	7	10	1	18	5.9	68.4	m	n	ae	n	S	S	N	N	N	6	2	8
Mycobacterium tuberculosis H37Ra	2	2	3	7	4.4	65.6	m	n	ae	n	S	H	O	A	P	6	3	9
Mycobacterium tuberculosis H37Rv	2	2	3	7	4.4	65.6	m	n	ae	n	S	H	O	A	P	6	3	9
Mycobacterium ulcerans Agy99	3	2	0	5	5.8	65.4	m	n	ae	n	S	H	O	A	P	6	3	9
Mycobacterium vanbaalenii PYR-1	11	16	3	30	6.5	67.8	m	n	ae	n	S	S	N	N	N	6	2	8
Nocardia farcinica	14	13	1	28	6.3	70.7	m	n	ae	y	P	M	F	A	P	6	3	9
Nocardioides sp. JS614	14	11	2	27	5.3	71.5	m	n	ae	n		S	N	N	N	4	3	7
Propionibacterium acnes KPA171202	6	0	0	6	2.6	60.0	m	n	an	n		H	O	A	F/P	3	2	5
Renibacterium salmoninarum ATCC 33209	4	5	0	9	3.2	56.3	m	n	f	n	S	M	F	A	P	3	2	5
Rhodococcus erythropolis PR4	20	12	1	33	6.9	62.3	m	n	ae	n	P	W	N	N	N	7	2	9
Rhodococcus jostii RHA1	17	24	16	57	9.7	67.0	m	n	ae	n	P	S	N	N	N	7	4	11
Rhodococcus opacus B4	17	23	10	50	8.8	67.6	m	n	ae	n	P		N	N	N	7	3	10
Rubrobacter xylanophilus DSM 9941	8	4	1	13	3.2	70.5	t	n	ae	n	SP	S	N	N	N	6	3	9
Saccharopolyspora erythraea	23	28	1	52	8.2	71.1	m	n	ae	y	P	S	N	N	N	12	4	16
Salinispora arenicola CNS-205	7	13	0	20	5.8	69.5	m	y	ae	y		W	N	N	N	6	3	9
Salinispora tropica CNB-440	9	9	0	18	5.2	69.5	m	n	ae	y	SP	W	N	N	N	7	3	10
Streptomyces avermitilis MA-4680	32	18	0	50	9.1	70.7	m	n	ae	y	P	S	N	N	N	10	3	13
Streptomyces coelicolor A3(2)	45	24	2	71	9.1	72.0	m	n	ae	y	P	S	N	N	N	10	4	14
Streptomyces griseus subsp. *griseus* NBRC 13350	36	11	1	48	8.6	72.2	m	n	ae	y	P	S	N	N	N	7	4	11
Thermobifida fusca YX	11	4	1	16	3.6	67.5	t	n	ae	y	P	M	F	A	P	7	2	9

TºC: m= mesophile, t=termophile; Motility: n=no, y=yes; OxyReq: an=anaerobe, f=facultative, ae=aerobe; Spor: n=no, y=yes; CellArr: S= single cells, P= pluricellular forms (including filamentous forms), SP= single or pluricellular forms; Hab: S= soil (or sediments), w=water, M=multiple, H=host; HostDep: N=none, F=facultative, O=obligate; Host: N=none, A=Animal, P=Plant; BioRel: N=none, F=part of the organisms normal flora, P=pathogenic, FP=part of the organisms normal flora/opportunistic pathogen; #T+PK+NRP, number of genetic pathways each organism has assigned in KEGG under the categories "1.9 Metabolism of Terpenoids and Polyketides"; #other sec met, number of genetic pathways each organism has assigned in KEGG under the categories "1.10 Biosynthesis of Other Secondary Metabolites"; total sec met, sum of the two previous variables.

Ordinal variables' codes:

Frequency of REC+LuxR, LuxR, other and SeqTotal	Code
[0-5]	1
]5-10]	2
]10-15]	3
]15-20]	4
]20-25]	5
]25-30]	6
]30-35]	7
]35-40]	8
]40-45]	9
]45-50]	10
]50-55]	11
]55-60]	12
]60-65]	13
]65-70]	14
]70-75]	15
]75-80]	16
]80-85]	17
]85-90]	18
]90-95]	19
>95	20

GenSize	Code
[0-1]	1
]1-2]	2
]2-3]	3
]3-4]	4
]4-5]	5
]5-6]	6
]6-7]	7
]7-8]	8
]8-9]	9
]9-10]	10
]10-11]	11

OxyReq	Code
na	1
f	2
ae	3

Spor	Code
n	1
y	2

CellArr	Code
S	1
SP	2
P	3

%G+C	Code
[50-55]	1
]55-60]	2
]60-65]	3
]65-70]	4
]70-75]	5

TºC	Code
m	1
t	2

Mot	Code
n	1
y	2

HostDep	Code
N	0
F	1
O	2

Host	Code
P	1
A	2
