# Supplementary material for: A Walk into the LuxR Regulators of Actinobacteria: Phylogenomic Distribution and Functional Diversity
Source: PLoS One. 2012 Oct 8;7(10):e46758. doi: 10.1371/journal.pone.0046758 (PMC3466318; doi:10.1371/journal.pone.0046758)
Supplement: Table S2 — Multiple regression analysis of the variables considered in this study regarding the distribution of the total number of LuxR regulators among the actinobacteria considered. (PDF) [file pone.0046758.s005.pdf]

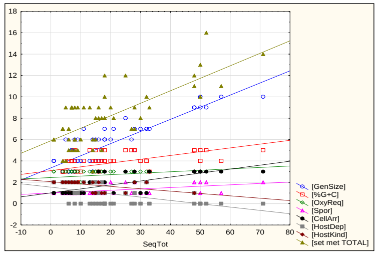

	b*	Std.Err.	b	Std.Err.	t(18)	p-value	Valid
Intercept			34.8175	13.27688	2.62241	0.017265	
[GenSize]	0.579813	0.148677	4.3795	1.12300	3.89983	0.001050	53
[%G+C]	-0.103206	0.110975	-1.5271	1.64206	-0.93000	0.364678	53
[OxyReq]	-0.246033	0.102070	-6.2206	2.58070	-2.41044	0.026845	53
[Spor]	-0.298529	0.122980	-11.2459	4.63280	-2.42746	0.025921	51
[CellArr]	0.284278	0.126910	5.3208	2.37535	2.23999	0.037950	45
[HostDep]	-0.104381	0.081808	-1.8322	1.43595	-1.27593	0.218201	53
[HostKind]	-0.383794	0.105841	-15.1397	4.17517	-3.62613	0.001932	29
[set met TOTAL]	0.325103	0.174351	1.9831	1.06354	1.86465	0.078622	53

	Value
Multiple R	0.960722248
Multiple R²	0.922987238
Adjusted R²	0.888759344
F(8,18)	26.96593699
p	1.69034E-08
Std.Err. of Estimate	5.431112637

The Multiple Regression Analysis was made using the total number of sequences containing a LuxR domain as the dependent variable. The independent variables considered were the ordinal scales of GenSize, %G+C, OxyReq, Spor, CellArr, HostDep and HostKind, and the original metric scale of the total number of secondary metabolic pathways. Statistical significant values (α<0.05) are highlighted in red.

%G+C

	Value
Multiple R	0.524898993
Multiple R²	0.275518953
Adjusted R²	0.261313443
F(1,51)	19.39521631
p	5.46015E-05
Std.Err. of Estimate	13.86526555

	b*	Std.Err.	b	Std.Err.	t(51)	p-value	Valid
Intercept			-10.4640	6.890179	-1.51868	0.135018	
[%G+C]	0.524899	0.119187	7.7668	1.763576	4.40400	0.000055	53

	Value
Multiple R	0.860906774
Multiple R²	0.741160474
Adjusted R²	0.730806893
F(2,50)	71.58493958
p	2.11722E-15
Std.Err. of Estimate	8.370088724

	b*	Std.Err.	b	Std.Err.	t(50)	p-value	Valid
Intercept			-19.7283	4.272580	-4.61743	0.000027	
[%G+C]	0.089499	0.085349	1.3243	1.262880	1.04863	0.299392	53
[GenSize]	0.809453	0.085349	6.1140	0.644662	9.48409	0.000000	53

	Value
Multiple R	0.524947696
Multiple R²	0.275570084
Adjusted R²	0.246592887
F(2,50)	9.509894535
p	0.000316162
Std.Err. of Estimate	14.00273764

	b*	Std.Err.	b	Std.Err.	t(50)	p-value	Valid
Intercept			-10.7683	8.641036	-1.24618	0.218504	
[%G+C]	0.518976	0.156300	7.6791	2.312729	3.32038	0.001684	53
[OxyReq]	0.009285	0.156300	0.2348	3.951848	0.05941	0.952866	53

	Value
Multiple R	0.591090612
Multiple R²	0.349388111
Adjusted R²	0.322279283
F(2,48)	12.88835144
p	3.30924E-05
Std.Err. of Estimate	13.28578737

	b*	Std.Err.	b	Std.Err.	t(48)	p-value	Valid
Intercept			-16.6098	7.226986	-2.29830	0.025943	
[%G+C]	0.375908	0.132769	5.5622	1.964549	2.83129	0.006754	53
[Spor]	0.309948	0.132769	11.6761	5.001567	2.33449	0.023802	51

	Value
Multiple R	0.749474854
Multiple R²	0.561712557
Adjusted R²	0.540841726
F(2,42)	26.91376148
p	2.99875E-08
Std.Err. of Estimate	10.95022282

	b*	Std.Err.	b	Std.Err.	t(42)	p-value	Valid
Intercept			-11.8266	5.911233	-2.00071	0.051915	
[%G+C]	0.176687	0.121888	2.6144	1.803538	1.44959	0.154600	53
[CellArr]	0.638314	0.121888	11.9472	2.281344	5.23691	0.000005	45

	Value
Multiple R	0.655172356
Multiple R²	0.429250816
Adjusted R²	0.406420848
F(2,50)	18.80207751
p	8.14953E-07
Std.Err. of Estimate	12.42903506

	b*	Std.Err.	b	Std.Err.	t(50)	p-value	Valid
Intercept			4.32892	7.375456	0.58694	0.559888	
[%G+C]	0.385179	0.113422	5.69940	1.678272	3.39599	0.001348	53
[HostDep]	-0.416237	0.113422	-7.30613	1.990870	-3.66982	0.000589	53

	Value
Multiple R	0.601900734
Multiple R²	0.362284494
Adjusted R²	0.313229455
F(2,26)	7.385265638
p	0.002885032
Std.Err. of Estimate	13.47682672

	b*	Std.Err.	b	Std.Err.	t(26)	p-value	Valid
Intercept			28.3531	22.53697	1.25807	0.219547	
[%G+C]	0.298901	0.197398	4.4228	2.92084	1.51421	0.142037	53
[HostKind]	-0.371269	0.197398	-14.6457	7.78686	-1.88082	0.071242	29

	Value
Multiple R	0.724654569
Multiple R²	0.525124245
Adjusted R²	0.506129215
F(2,50)	27.6453493
p	8.21296E-09
Std.Err. of Estimate	11.33717269

	b*	Std.Err.	b	Std.Err.	t(50)	p-value	Valid
Intercept			-20.5751	5.969136	-3.44692	0.001158	
[%G+C]	0.156516	0.121083	2.3159	1.791635	1.29264	0.202079	53
[set met TOTAL]	0.620734	0.121083	3.7865	0.738606	5.12651	0.000005	53

Host Kind

	Value
Multiple R	0.545531517
Multiple R²	0.297604636
Adjusted R²	0.283832178
F(1,51)	21.60867967
p	2.39568E-05
Std.Err. of Estimate	13.65228972

	b*	Std.Err.	b	Std.Err.	t(51)	p-value	Valid
Intercept			27.91237	2.728698	10.22919	0.000000	
[HostDep]	-0.545532	0.117356	-9.57560	2.059928	-4.64851	0.000024	53

	Value
Multiple R	0.862223076
Multiple R²	0.743428633
Adjusted R²	0.733165779
F(2,50)	72.43877633
p	1.69005E-15
Std.Err. of Estimate	8.333335314

	b*	Std.Err.	b	Std.Err.	t(50)	p-value	Valid
Intercept			-12.5192	4.646470	-2.69434	0.009578	
[HostDep]	-0.106763	0.085716	-1.8740	1.504563	-1.24553	0.218741	53
[GenSize]	0.798963	0.085716	6.0348	0.647440	9.32100	0.000000	53

	Value
Multiple R	0.564103013
Multiple R²	0.318212209
Adjusted R²	0.290940698
F(2,50)	11.66830111
p	6.93794E-05
Std.Err. of Estimate	13.58436597

	b*	Std.Err.	b	Std.Err.	t(50)	p-value	Valid
Intercept			16.32501	9.808904	1.66430	0.102307	
[HostDep]	-0.486461	0.126272	-8.53876	2.216422	-3.85250	0.000334	53
[OxyReq]	0.155232	0.126272	3.92483	3.192622	1.22935	0.224696	53

	Value
Multiple R	0.640051923
Multiple R²	0.409666464
Adjusted R²	0.385069234
F(2,48)	16.65498325
p	3.20877E-06
Std.Err. of Estimate	12.65537527

	b*	Std.Err.	b	Std.Err.	t(48)	p-value	Valid
Intercept			9.59122	6.594518	1.45442	0.152337	
[HostDep]	-0.433443	0.116951	-7.60814	2.052811	-3.70621	0.000544	53
[Spor]	0.353023	0.116951	13.29881	4.405669	3.01857	0.004058	51

	Value
Multiple R	0.786132531
Multiple R²	0.618004356
Adjusted R²	0.599814087
F(2,42)	33.97444887
p	1.67196E-09
Std.Err. of Estimate	10.22286552

	b*	Std.Err.	b	Std.Err.	t(42)	p-value	Valid
Intercept			3.86908	4.618112	0.83781	0.406883	
[HostDep]	-0.304063	0.103684	-5.33716	1.819936	-2.93261	0.005422	53
[CellArr]	0.615391	0.103684	11.51815	1.940623	5.93528	0.000000	45

	Value
Multiple R	0.693237952
Multiple R²	0.480578859
Adjusted R²	0.440623386
F(2,26)	12.02786076
p	0.000200334
Std.Err. of Estimate	12.16281124

	b*	Std.Err.	b	Std.Err.	t(26)	p-value	Valid
Intercept			57.2987	10.25118	5.58948	0.000007	
[HostDep]	-0.432187	0.146220	-7.5861	2.56658	-2.95572	0.006552	53
[HostKind]	-0.442517	0.146220	-17.4562	5.76804	-3.02637	0.005520	29

	Value
Multiple R	0.763725367
Multiple R²	0.583276436
Adjusted R²	0.566607493
F(2,50)	34.9918079
p	3.13474E-10
Std.Err. of Estimate	10.62034911

	b*	Std.Err.	b	Std.Err.	t(50)	p-value	Valid
Intercept			-5.22719	6.045389	-0.86466	0.391356	
[HostDep]	-0.299608	0.100501	-5.25721	1.764065	-2.98017	0.004439	53
[set met TOTAL]	0.588387	0.100501	3.58916	0.613052	5.85457	0.000000	53

Total number of secondary metabolic pathways

	Value
Multiple R	0.713620902
Multiple R²	0.509254791
Adjusted R²	0.499632336
F(1,51)	52.92358211
p	2.00359E-09
Std.Err. of Estimate	11.4114994

	b*	Std.Err.	b	Std.Err.	t(51)	p-value	Valid
Intercept			-16.4551	5.080026	-3.23917	0.002112	
[set met TOTAL]	0.713621	0.098094	4.3531	0.598373	7.27486	0.000000	53

	Value
Multiple R	0.857718508
Multiple R²	0.735681039
Adjusted R²	0.72510828
F(2,50)	69.58269615
p	3.57444E-15
Std.Err. of Estimate	8.458218946

	b*	Std.Err.	b	Std.Err.	t(50)	p-value	Valid
Intercept			-16.3543	3.765354	-4.34338	0.000069	
[set met TOTAL]	-0.027005	0.134510	-0.1647	0.820508	-0.20076	0.841697	53
[GenSize]	0.880314	0.134510	6.6493	1.015989	6.54462	0.000000	53

	Value
Multiple R	0.721278068
Multiple R²	0.520242052
Adjusted R²	0.501051734
F(2,50)	27.10961092
p	1.06061E-08
Std.Err. of Estimate	11.39530235

	b*	Std.Err.	b	Std.Err.	t(50)	p-value	Valid
Intercept			-11.3807	6.944092	-1.63890	0.107512	
[set met TOTAL]	0.791216	0.121874	4.8264	0.743431	6.49208	0.000000	53
[OxyReq]	-0.130416	0.121874	-3.2974	3.081432	-1.07009	0.289719	53

	Value
Multiple R	0.715348257
Multiple R²	0.511723129
Adjusted R²	0.49137826
F(2,48)	25.15244085
p	3.37278E-08
Std.Err. of Estimate	11.50957736

	b*	Std.Err.	b	Std.Err.	t(48)	p-value	Valid
Intercept			-17.4389	5.592036	-3.11853	0.003070	
[set met TOTAL]	0.672924	0.130377	4.1048	0.795300	5.16136	0.000005	53
[Spor]	0.064223	0.130377	2.4194	4.911460	0.49259	0.624545	51

	Value
Multiple R	0.823615072
Multiple R²	0.678341788
Adjusted R²	0.66302473
F(2,42)	44.28669
p	4.52155E-11
Std.Err. of Estimate	9.380819175

	b*	Std.Err.	b	Std.Err.	t(42)	p-value	Valid
Intercept			-19.1493	4.568189	-4.19188	0.000139	
[set met TOTAL]	0.444745	0.104561	2.7129	0.637821	4.25346	0.000115	53
[CellArr]	0.491306	0.104561	9.1957	1.957043	4.69875	0.000028	45

	Value
Multiple R	0.810011275
Multiple R²	0.656118266
Adjusted R²	0.629665825
F(2,26)	24.80369447
p	9.40413E-07
Std.Err. of Estimate	9.896433476

	b*	Std.Err.	b	Std.Err.	t(26)	p-value	Valid
Intercept			16.5891	11.56752	1.43411	0.163459	
[set met TOTAL]	0.612083	0.118974	3.7337	0.72574	5.14470	0.000023	53
[HostKind]	-0.396451	0.118974	-15.6390	4.69322	-3.33226	0.002591	29
