# Supplementary material for: A Walk into the LuxR Regulators of Actinobacteria: Phylogenomic Distribution and Functional Diversity
Source: PLoS One. 2012 Oct 8;7(10):e46758. doi: 10.1371/journal.pone.0046758 (PMC3466318; doi:10.1371/journal.pone.0046758)

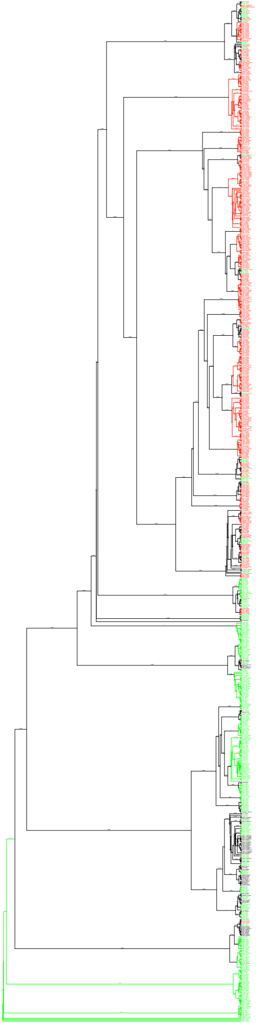

Supplement: Figure S2 — Maximum-likelihood tree of all LuxR-containing sequences retrieved from Actinobacteria. Sequences are indicated by their locus tag followed by a letter code corresponding to their domain composition (L, LuxR; RL, REC+LuxR; CL, CHD+LuxR; HL, HDc+LuxR; AL, AAA+LuxR; PL, Pkc+LuxR; FL, FHA+LuxR; TL, TRP+LuxR; LC, LuxR+ CSP_CDS; PsL, PAS+LuxR; PTL, Pkc+ TRP+LuxR). A colour-code is used to distinguish between the two main subfamilies: single-domain LuxR proteins are in green, whereas REC+LuxR proteins are in red. Numbers in the branches correspond to the SH-like interpretation of the aLRT values. (PDF) [file pone.0046758.s002.pdf]
